# Dog assisted education in children with rheumatic diseases and adolescents with chronic pain in Germany

**DOI:** 10.3205/zma001626

**Published:** 2023-06-15

**Authors:** Jan Kiesewetter, Nadja Herbach, Iris Landes, Julia Mayer, Verena Elgner, Karin Orle, Alexandra Grunow, Rovena Langkau, Christine Gratzer, Annette F. Jansson

**Affiliations:** 1 Klinikum der LMU München, Institute for Medical Education, Munich, Germany; 2 Munich, Germany; 3 Ulm, Germany; 4 eo ipso Strategie & Entwicklung GmbH, Krailling, Germany; 5 K-9 Headquarter, Pürgen, Germany; 6 Ludwig-Maximilians-University, Dr. von Hauner Children's Hospital, Department of Rheumatology & Immunology, Munich, Germany

**Keywords:** animal assisted intervention, dog assisted education, chronic pain, rheumatic diseases, children, adolescents

## Abstract

**Objectives:**

Animal assisted intervention is an increasingly accepted tool to improve human well-being. The present study was performed to assess whether dog assisted education has a positive effect on children suffering from rheumatic disorders with pain and adolescents with chronic pain syndrome.

**Design:**

Two groups of juvenile patients were recruited: 7-17-year-old children in children with rheumatic diseases and adolescents with chronic pain syndromes. Overall, n=26 participated in the intervention, and n=29 in the control group.

**Setting:**

The intervention group met once a month, 12 times overall, for working with man trailing dogs in various locations.

**Main outcome measures:**

The influence of dog assisted education on quality of life (PedsQL^TM^ Scoring Algorithm), pain intensity, perception, coping (Paediatric Pain Coping Inventory-Revised), and state anxiety (State Trait Anxiety Inventory) was assessed.

**Results:**

The quality of life increased significantly in the investigated period, but for both, the intervention and the control group. The state anxiety of children was lower after the dog assisted education than before. After the dog training sessions, state anxiety was 18% to 30% lower than before the intervention.

Some participants noted subjectively improved pain coping and changes in pain perception, which were not found in the data.

**Conclusion:**

Our results indicate that for children with rheumatic diseases and adolescents with chronic pain syndromes dog assisted education (1) might lead to an increase of the quality of life, (2) leads to decreased state anxiety from pre to post intervention and (3) does not influence pain perception, frequency and intensity.

## Zusammenfassung

**Zielsetzung:**

Tiergestützte Interventionen sind ein zunehmend akzeptiertes Instrument zur Verbesserung des menschlichen Wohlbefindens. Die vorliegende Studie wurde durchgeführt, um herauszufinden, ob tiergestützte Erlebnispädagogik positive Effekte bei Kindern mit entzündlich-rheumatischen Erkrankungen sowie auf Jugendliche mit chronischem Schmerzsyndrom hat.

**Studiendesign:**

Es wurden zwei Gruppen von Patienten im Alter von 7-17 Jahren rekrutiert: Kinder mit rheumatischen Erkrankungen und Jugendliche mit chronischen Schmerzsyndromen. Insgesamt nahmen n=26 an den Interventionsgruppen und n=29 an den Kontrollgruppen teil.

**Rahmenbedingungen:**

Die Interventionsgruppe traf sich einmal im Monat, insgesamt 12 Mal, um an verschiedenen Orten mit Mantrailing-Hunden zu arbeiten.

**Wichtigste gemessene Parameter:**

Der Einfluss der tiergestützten Pädagogik auf Lebensqualität (PedsQL^TM^ Scoring Algorithm), Schmerzintensität, -wahrnehmung, -bewältigung (Paediatric Pain Coping Inventory-Revised) und Angstzustand (State Trait Anxiety Inventory) wurde untersucht.

**Ergebnisse:**

Im Untersuchungszeitraum zeigte sich eine signifikante Verbesserung der Lebensqualität sowohl in der Interventions- als auch in der Kontrollgruppe. Der Angstzustand der Kinder war nach den Interventionen geringer als vorher. Nach den Trainingssitzungen mit den Hunden war der Angstzustand um 18 bis 30% niedriger als vor der Intervention.

Einige Teilnehmer berichteten von einer subjektiv verbesserten Schmerzbewältigung und einer veränderten Schmerzwahrnehmung, die sich in den Daten nicht nachweisen ließ.

**Fazit:**

Unsere Ergebnisse deuten darauf hin, dass die hundegestützte Pädagogik (1) bei Kindern mit rheumatischen Erkrankungen und Jugendlichen mit chronischen Schmerzsyndromen zu einer Steigerung der Lebensqualität führen kann (2), zu einer Verringerung der Angstzustände vor und nach der Intervention führt, (3) und die Schmerzwahrnehmung, -häufigkeit und -intensität nicht beeinflusst.

## Background

The involvement of animals in healthcare, namely animal-assisted-intervention (AAI), has gained increasing attention in recent years. Animals accompany children and adults in different conditions, with the intention of improving distress in a structured manner with a specific goal [1]. AAI was implemented in treatment of psychiatric disorders, in pain management as well as for alleviating stress and elevating mood. AAI is further categorized into animal-assisted-therapy (AAT), animal-assisted-education (AAE) and animal-assisted-activity (AAA). The difference between these types of interventions comprise the type of specialisation and expertise of the persons conducting the intervention, the focus of the intervention and the obligation of documentation [1]. To our knowledge, the term animal assisted *therapy* is frequently used but defined standards and controlled studies for indications are lacking [2]. 

It is important to distinguish dog-ownership, companion-animals, animals assisting patients, and animal assisted interventions, specifically trained animals and trainers with specifically designed treatment with animals in order to enlarge the empirical basis for AAT, AAE, AAI and AAA. For example, AAI has proven effective for patients with chronic pain [3], yet companion-animals show mixed results [3]. 

A plethora of animals have been used in AAI, including dogs, horses, dolphins, cats, cows, rabbits, ferrets, guinea pigs and birds [3]. AAI resulted in positive outcomes in different populations and diseases [4]. For example, interaction with dolphins significantly improved symptoms of depression in adults [5]. Furthermore, AAA using guinea pigs had significant impact on social functioning, skills and approaching behaviour in children with ASD [6]. For children and adolescents self-reported pain decreased after a short AAA intervention with dogs [1]. For acute care, pain decrease and health benefits for children and adolescents have been shown through AAT [1]. However, to increase the quality of life in adults and children with chronic pain has been suggested [1] but thus far not systematically studied. A reason for this lack of studies might be the fear of zoonoses and hygienic aspects, especially in stationary settings. 

Dogs are an attractive animal of choice for AAI, since they are a common companion animals that react on emotions of humans and show friendly, caring behaviour [1]. In addition, the interaction between dog and human leads to increased release of various hormones and substances associated with well-being or anxiety (beta-endorphin, oxytocin, prolactin, phenyl acetic acid, dopamine) in both, humans and dogs [7]. Consequentially, a recent study revealed, that children with pet dogs show a decreased probability of childhood anxiety and children with disabilities, learned to cope with anxiety [8]. 

For patients with chronic pain complementary therapy dog visits in a waiting room, temporarily reduced distress but did not lower pain in most patients [9]. In hospital settings a 15-minute therapy sessions with a dog led to lower pain levels of children that underwent surgery [10]. 

Patients with lasting and chronic pain have an increased sensitivity towards pain and disturbed rewarding stimuli induced by impaired operant learning [11]. Pain relief is usually experienced rewarding and attenuates sensitivity to pain. Subjects with chronic pain cannot process rewards adequately. Operant learning induces a generally increased sensitivity to pain and pain responses as well as pain behaviour. This leads to a vicious circle with maintenance of chronic pain. “Re-learning” of attenuated pain sensitivity and decreased pain behaviour is difficult [11]. However, recent studies show a pain- and emotional distress-reducing effect of AAI also in patients with chronic pain [12], [13]. However, to our knowledge, no study has studied effects of AAI on children and adolescents with chronic pain. As we use dogs in our study, but don’t study therapeutic but rather quality of life effects we use the specific AAI term of dog-assisted education (DAE). Understandably, shorter interventions closer to a stationary setting are much more common and thus more commonly studied. The program for the participants of the present study is complex and elaborate but we hypothesized the beneficial effects for the quality of life, chronic pain intensity and coping and state and trait anxiety could be equally grand and worth the effort.

Our research question is *whether children and adolescents with chronic pain would profit from DAE in a qualified setting*. 

We hypothesized that

1. DAE increases quality of life of children and adolescents with chronic pain,
2. DAE improves pain coping over the time course of the intervention and
3. DAE reduces anxiety from before to after the intervention.

## Methods

### Study design

The study was a prospective non-randomized, controlled study, conducted between May 2013 and November 2016 in an outpatient setting. 

Two age-groups of patients (children, age: 7-11; adolescents, age: 12-18) with chronic inflammatory disorder and/or pain syndrome were included in the study (see participants). We chose two age groups because of different interaction styles with the animals. Children interact with the animals and the parents, the adolescents interact with the animals without any parental support. Both age-groups consisted of an intervention group and a control group. Demographic data are given in table 1 (Tab. 1). The 11 intervention days were scheduled in 4-week intervals and took place in the afternoon (see table 2 (Tab. 2)). The group met 12 times overall. Control group was a matched control-group regarding age, sex, and diagnosis and were assigned to the control group because of too long travel distance. Control and intervention group filled out the questionnaire at the same time points. To maximize power for the study we report the results for the measures that were available in both children and adolescents, compared to a combined control-group. 

Locations and goals of the interventions were set up during concept meetings by experts for experience-based learning (VE, KO), dog trainers (AG, RL) and a paediatrician (AJ) before beginning the study. 

Both self-reported and parental assessment questionnaires were used. The different questionnaires and investigated time-points are summarized in table 2 (Tab. 2) and detailed below.

Approval of the studies was obtained from the LMU ethics committee (214-13; 393-15).

### Intervention

The twelve interventions were performed in monthly intervals and were conducted by eight to ten certified dog trainers and their approved man trailing dogs from the K-9 Suchhundezentrum (AG, RL). The dogs were variable in size and character, from e.g. dachshunds (≈5kg) to bloodhounds (≈40kg). Participants could choose the dog they wanted to work with. Interventions took place during the week in the afternoon and lasted about three hours. Parents accompanied children of the younger age group but did not take part in the intervention itself. For details of the intervention see attachment 1 .

### Participants

Participants with chronic inflammatory disorders (rheumatic diseases, children) and chronic pain syndrome (adolescents) were *included* in the study. Chronic inflammation happens when the response of the body to fight against infections, injuries etc. and causes abdominal pain, chest main, rashes, fever, fatigue. Chronic pain refers to pain that continues long (<3-6 months) after the cause is gone. Participants were *excluded* if current medication was modified in the last three months or change of medication was planned during the study. Participants were diagnosed the Department of Rheumatology & Immunology, Dr. von Hauner Childrens Hospital, Munich, and were patients from practices and hospitals in and around Munich. For the study 130 service users were approached. Parents of eligible patients were contacted by phone. Parents and children were informed and gave written consent. Some participants were dog owners or with other pets and animals (e.g. cats, guinea pigs, hamsters). Pet ownership is shown in table 1 (Tab. 1). Exact diagnoses are shown in the attachment 1 , supplement 1.

## Assessment tools

Questionnaires were chosen according to the main outcomes to quantify quality of life, pain coping and state anxiety. The German version of the questionnaires were used.

### PedsQLTM scoring algorithm (scoring the pediatric quality of life inventory) 

The total scale score of the PedsQL was used to verify the hypothesis that DAE increases quality of life of children and adolescents with chronic pain. The sum of all items related to the number of items answered creates the total scale score [14].

The PedsQL was filled out by both parents (parental-assessment) and children/adolescents (self-assessment) before and at 7 time-points during the intervention (see table 2 (Tab. 2)). 

### PPCI-R (Paediatric Pain Coping Inventory-Revised)

The PPCI-R was implemented to verify the hypothesis that DAE improves pain coping over the time course of the study [15]. We used the PPCI-R total score, calculated from all items. Participants answered the PPCI-R questionnaire before starting and right after finishing the intervention program (see table 2 (Tab. 2)). 

### STAI(C) (State-Trait-Anxiety Inventory)

State anxiety inventory is a validated and reliable test and was implemented to verify the hypothesis that the interventions reduce anxiety from before to after the intervention. The STAI [16] is a self-assessment questionnaire that describes anxiety as a state at present (state) and in general (trait) in children (STAIC) and adolescents (STAI). 

The STAI-S was implemented before and immediately after every dog training (intervention group only) at twelve and six time-points in children and adolescents, respectively. 

### Additional questionnaires

Additional questionnaires, their background and explanation, are listed in attachment 1 , supplement 2.

### Statistics

Statistical analyses were performed using IBM SPSS Statistics 24.0. Frequencies of main scores were calculated, Normal distribution was checked. Repeated measures analysis was implemented for all questionnaires. Primary outcome was the main parameter of the questionnaire with group as between-participants effect. Missing values are replaced by means of the remaining participants. Follow-up Bonferroni tests were performed for pairwise comparisons. Statistical analysis of the correlations were carried out using Pearson’s correlation test (PedsQL). Paired Student’s T-Tests were used for comparing pre-post conditions (STAI-S). 

## Results

### Demographic data

Demographic data for both children and adolescents and overall are listed in table 1 (Tab. 1).

### PedsQLTM scoring algorithm 

Data are presented in table 3 (Tab. 3) for both the self- and parental assessment.

#### PedsQL self-assessment

A repeated measures ANOVA showed that the main effect for point of measured was significant. The total scale scores increased with time in the intervention and the control group (F(1;484)=38.00, p<.001; eta^2^=.49). The intervention group did not show overall increased scores than the control group (F(12;484)=1.09, p=35, n.s.). 

#### PedsQL parental assessment

The total scale scores increased with time in the intervention and the control group (F(1;12)=38.48; p<.001; eta^2^=.34). The interaction of time and group was significant (F(1;12)=2.082;p=0.02; eta^2^=.05). The post-hoc tests revealed that the significance is driven by two differences where once the control group had significantly lower values, at one time point significantly higher values than the intervention group. The significant differences are marked with an asterisk int 3. 

#### PedsQL self- versus parental assessment

There was some consensus of self-assessment and external assessment of the total health summary scales at assessments with a significant mean correlation of R=.17. A repeated measures ANOVA revealed that the self-assessed quality of life was rated significantly higher than the parental assessment (F(1;12)=6.77; p=.01 eta^2^=.02). The post-hoc tests revealed that the significance is driven by one significantly higher value of the self-assessment than the parental assessment at the fourth intervention. 

### PPCI-R 

The PPCI was implemented to verify the hypothesis that DAE improves pain coping over the time course of the study. The PPCI was not different comparing intervention and control groups (F(1;51)=.15; p=.69; n.s.). Repeated measures ANOVA did reveal changes of pain coping over time for both, the intervention and the control group (F(1;51)=109.83; p<.001; eta^2^=.68). Data are shown in table 4 (Tab. 4).

### STAI-S

#### State anxiety (STAI-S) 

**Figure 1:**
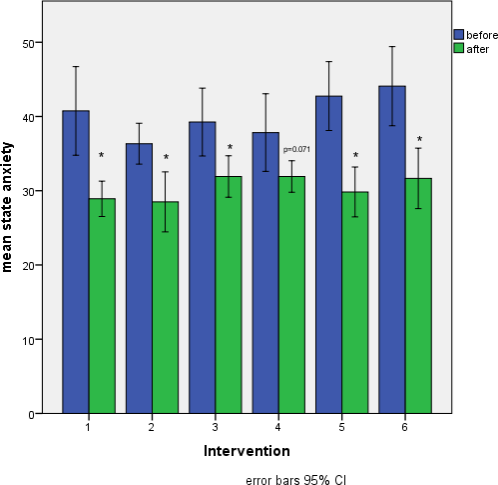
Development of state anxiety from pre to post intervention during the study.

A repeated measures ANOVA revealed a reduction of state anxiety from pre to post intervention (F(1;198)=31.20; p<.001; eta^2^=.14). A post-hoc comparison showed that the decline of state anxiety from pre-post intervention was significant for the first six interventions and ranged between 18% and 30% (see table 5 (Tab. 5)). From the seventh time point the state anxiety was lower before the intervention already (see figure 1 (Fig. 1)). 

## Discussion

Our research question was *whether children and adolescents with chronic pain would profit from DAE in a qualified setting*. We will separately present the results for pain and quality of life and anxiety.

### Pain and quality of life

DAE did not result in robust changes of pain perception, frequency, intensity. In other studies 15-minute therapy sessions with a dog led to lower pain levels of children that underwent total knee or hip arthroplasty [10]. The difference to our study could be explained by the three-week intervals between intervention and answering the questionnaire. AAI has a predominantly short-term effect, that can be measured right after the intervention but not or to a lesser extent before the next [4]. Furthermore, participants of the present study suffered from chronic pain, unlike the children that experienced acute pain induced by surgery [10], [17]. We could observe changes in quality of life over time but the score increased for both, intervention and control group. More research is needed to study whether the increased quality of life is for example more stable for the intervention than for the control group. Further studies could verify whether shorter interventions might also lead to the increased quality of life or whether the assessment alone might trigger a socially desired increase in quality of life.

#### Anxiety

In the present study we observed a decrease of state anxiety from before to after all interventions. This is in concordance with other studies, that also noted a pre-post effect of AAI but before the next session, STAI-S values were back to pre-intervention scores [4]. Interestingly, after half of the interventions we performed STAI-S levels were already at a lower level before the intervention, showing that the participants were more used to the dogs and the setting. A recent study revealed, that children with pet dogs in their home show a decreased probability of childhood anxiety [8] and children with physical and mental disabilities, learned to cope with anxiety [18]. 

It is well accepted, that chronic pain is associated with increased anxiety [19]. Patients suffering from rheumatic diseases and other chronic pain conditions also show high correlation of increased STAI scores with depression [20], [21]. Vice versa, over 80% of paediatric patients with chronic pain show a comorbid anxiety disorder. Depression and anxiety may lead to chronic pain and chronic pain may trigger anxiety and depression [22]. Conversely, adolescents and children participating in our study, depression scores were not that frequent (16% of adolescents,10% of children, attachment 1 , supplement 4).

To our knowledge, there are no validated standards concerning character qualifications and health examinations of dogs or other animals. It is known however, that the effectiveness is associated with the species and personality of the animals [23]. Subsequently, it would be helpful for finding common standards that might enable international research collaborations [23]. In our pilot project we wanted to satisfy such standards in an outpatient setting. As with our study this proved to be complex and costly. 

Further studies in an outpatient setting could easily reduce complexity and costs. Our suggestions would be to expand the design to a multi-center study in order to include more patients. The inclusion of patients with more diverse diagnoses with chronic pain could help increase patient numbers to advance knowledge on how the intervention works. We chose the three week rhythm of the interventions in order to be feasible for patients, parents and cooperation partners. It would be interesting to see how beneficial weekly interventions or even daily interventions could change the effects. In our study our idea was to maximize transfer of the effects to the home of the patients a longer follow-up to the study could therefore be beneficial in shorter intervention studies.

More research is needed to verify our effects and in our experience with a few changes concerning the setting it might be possible to expand our design to a multi-center study. 

### Limitations

Due to organisational reasons the study could not be randomized or blinded, the number of available participants for intervention and control group was rather small. Participants of the intervention group had to be willing to work with dogs. Therefore, the results may not transfer to people with an aversive attitude towards dogs. Participants of the control group were matched with regards to sex, age and diagnoses and part of the control group to due travel distance. A randomized control group was thus not part of the study and control group participants could vary in other covarying factors due to travel distance. As well, we cannot rule out that covarying factors like physical activity or enriched social contact and communication with other patients with relatable symptoms might have positively influenced or even caused our results. 

Furthermore, due to a lack of studies with comparable interventions and outcome parameters no a priori sample size could be calculated as the effects could not be estimated. 

The quantitative approach we took was meant to get an insight into how large the effects of the DAE could be. However, a qualitative approach i.e. systematic interviews with the adolescents, children and parents might have complemented the present study to verify what DAE really meant to the patients. 

## Conclusion

DAE leads to decreased state anxiety from pre to post intervention in children with rheumatic diseases and adolescents with chronic pain syndromes. Further, despite having a very small sample, our results indicate that it might lead to an increase of the quality of life. Pain perception, frequency and intensity are not influenced. We consider DAE a valuable alternative treatment method to lower anxiety that might have a positive influence on the lives of children and adolescents with chronic diseases. 

## Data

Data for this article are available from Open Data LMU [24]. Further information on the data files can be found in attachment 1 .

## Note

The author Nadja Herbach deceased in 2020.

## Acknowledgements

First of all, we want to thank our generous sponsor Edith-Haberland-Wagner-Stiftung, Munich. Without their continuous support we wouldn’t have been able to perform this study.

Furthermore, we would like to thank:

- Verein Kinder-Rheumahilfe München e.V., for organizing and managing the interventions
- Children, adolescents, their parents and families for participating in the study
- The K-9 dog trainers and experts for taking care of the participants and involving them in the interventions:
- Daniela Rottenwaller, Ina Rebel, Gusti Kittlinger, Karin Haeringer, Mareike Haase, Eduard Bichler, Eva Fuhrmann, Andrea Roehricht, Sonja Zietlow, Carmen Schäffler, Robert Jansson, Matthias Lang, Christiane Wildhirt, Anja Lehmann, Leila Badry.
- The Specialists for Pediatric and Adolescent Rheumatology for recruiting participants: Rainer Berendes, Landshut; Philipp Schoof, München; Gerd Ganser, Sendenhorst; Gert Reutter-Simon, Nürnberg; Susanna Späthling-Mestekemper, München.

## Competing interests

The authors declare that they have no competing interests. 

## Erratum

The translation of the title into German has been corrected. 

## Supplementary Material

Supplemental material

## Figures and Tables

**Table 1:**
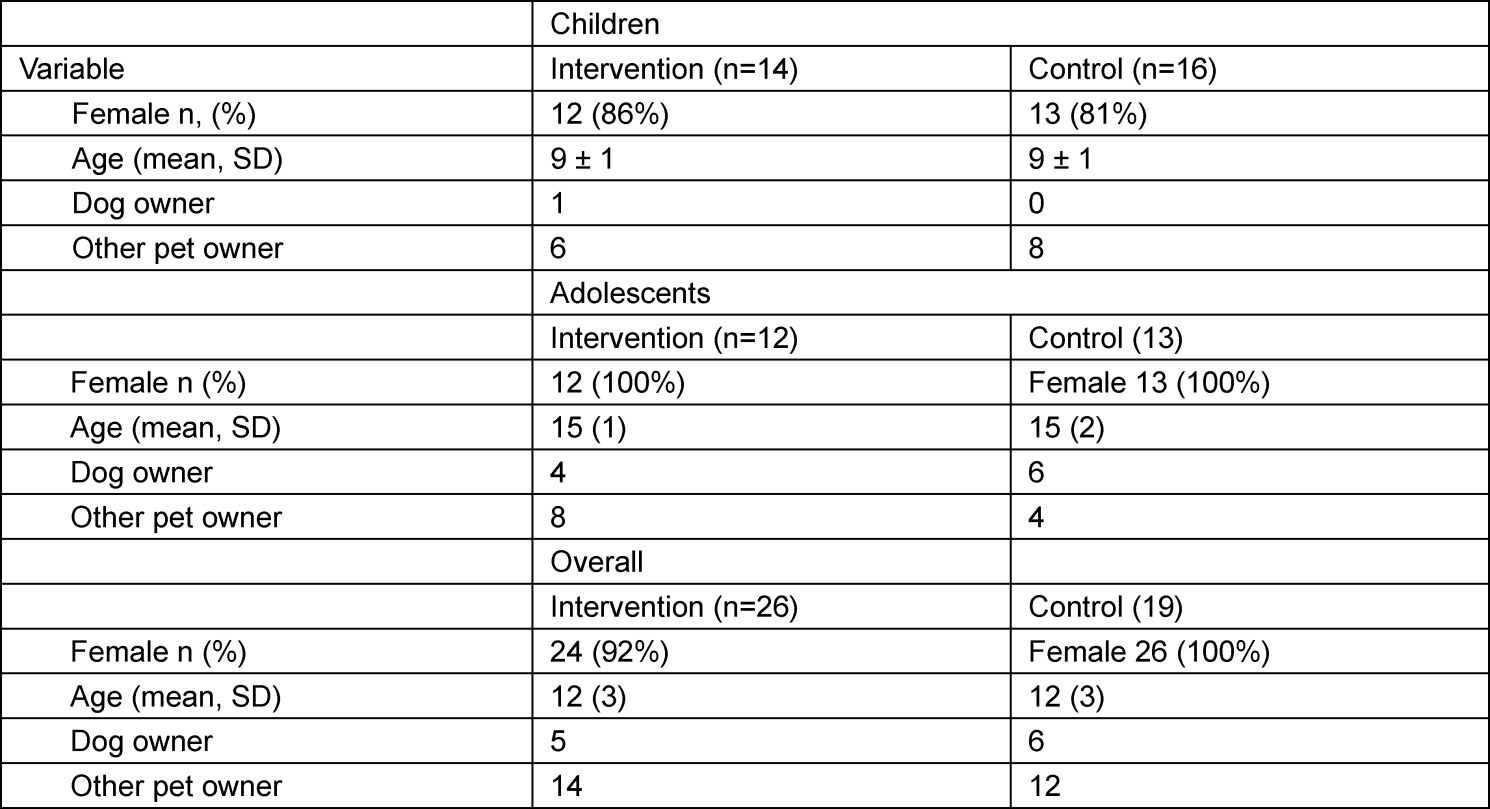
Demographic data

**Table 2:**
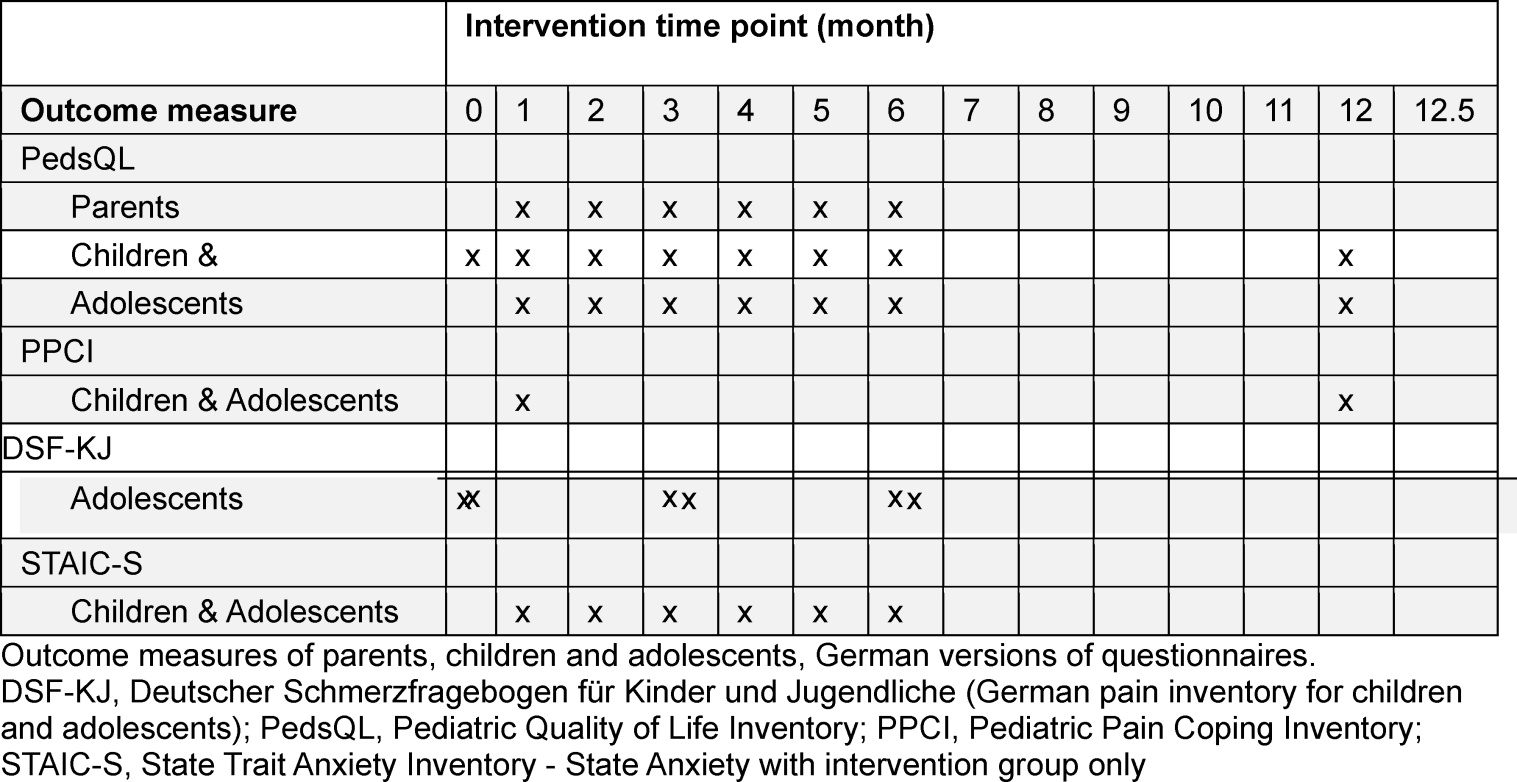
Study design/outcome measures

**Table 3:**
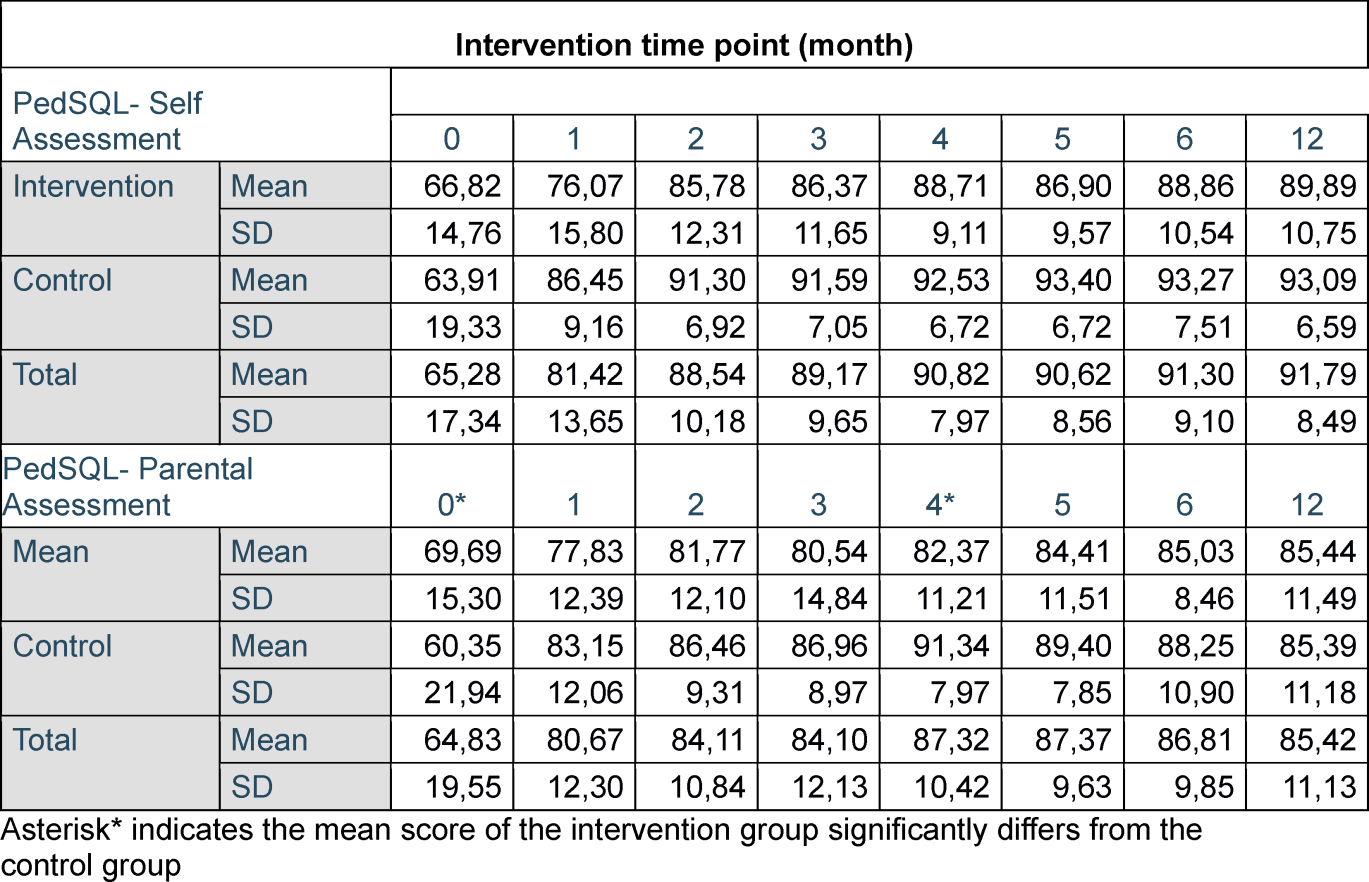
Descriptive statistics for PedsQL, self-assessment and parental assessment

**Table 4:**
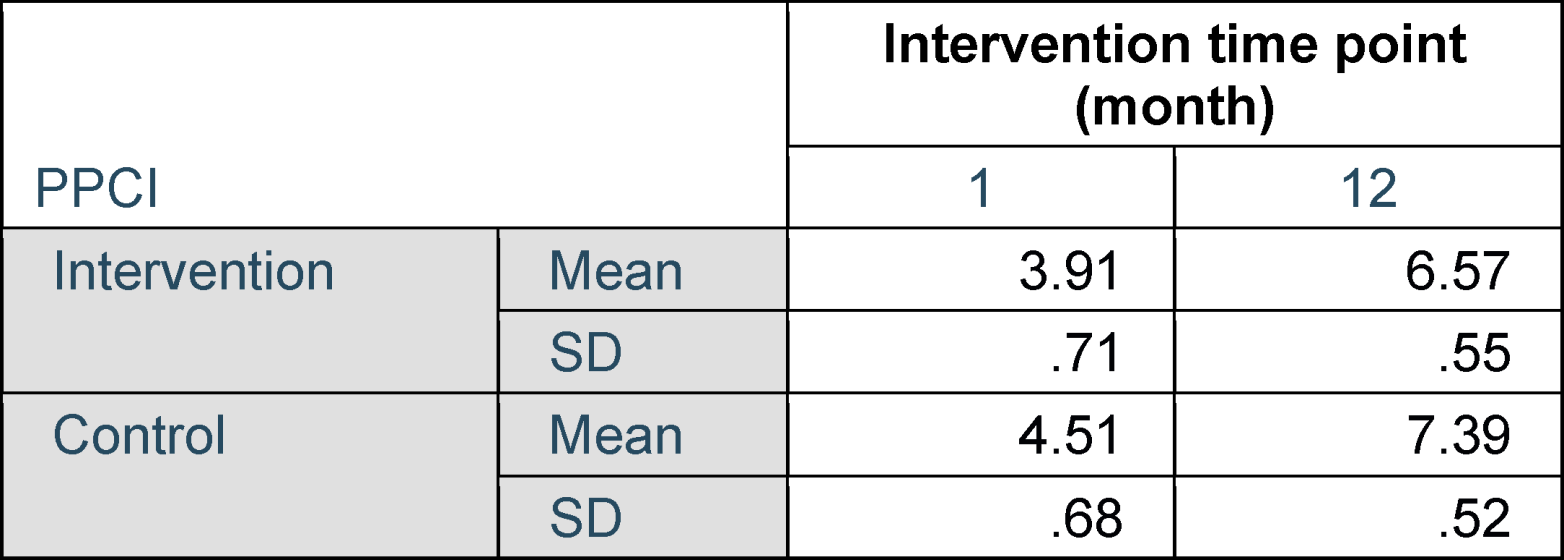
Descriptive statistics for PPCI, self-assessment

**Table 5:**
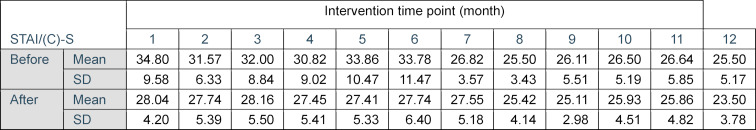
Descriptive statistics for STAI/C-S, self-assessment before and after the intervention
