# Supplementary material for: Dog assisted education in children with rheumatic diseases and adolescents with chronic pain in Germany
Source: GMS J Med Educ. 2023 Jun 15;40(4):Doc44. doi: 10.3205/zma001626 (PMC10407579; doi:10.3205/zma001626)
Supplement: Supplemental material [file JME-40-44-s-001.pdf]

Anhang 1: Ergänzendes Material

Details zur Intervention: Für die Interventionsgruppe fand die erste Veranstaltung auf einem Hundeübungsplatz des K-9 Suchhundezentrums statt. Die Teilnehmer wurden mit dem Team und untereinander bekannt gemacht. Zunächst lernten die Kinder/Jugendlichen etwas über die Bedürfnisse und das Verhalten von Hunden sowie den Umgang mit Mantrailing-Hunden. Danach wurden sie mit den Hunden bekannt gemacht, durften sie streicheln und an der Leine spazieren führen. Die zweite Intervention fand in einem ruhigen Bereich statt, wo die Kinder angeleitet wurden, eine Fährte zu legen, einen Mantrailing-Hund zu führen, dem Hund nach der Arbeit ein Leckerli zu geben und zu erfahren, wie die Kommunikation zwischen Hund und Mensch funktioniert. In der dritten Intervention bei Kindern ging es darum, zu lernen, wie Hunde ihre Emotionen in verschiedenen Situationen zeigen, zum Beispiel beim Stehpaddeln am Ammersee. Die nächste Intervention fand in einem Hochseilgarten statt, wo die Teilnehmer mit dem Hund den „Flying Fox" machen mussten. Die nächste Intervention fand am Münchner Flughafen statt, und die Teilnehmer hatten die Möglichkeit, als Hundetrainer zu arbeiten. Die nächste Intervention fand in einem Wald mit Unterstützung eines örtlichen Jägers statt. Er zeigte den Teilnehmern wichtige Merkmale des Waldes, z. B. die Spuren verschiedener Tiere. Intervention zehn fand in einem Altersheim statt. In der folgenden Intervention wurden die Teilnehmer angeleitet, die Hunde sicher zwischen den Gehegen eines Zoos zu führen. Die letzte Intervention fand auf dem Trainingsgelände des K-9 Suchhundezentrums statt, wo die Teilnehmer ihr Zertifikat erhielten. Die ersten beiden Interventionen bei der jugendlichen Gruppe waren die gleichen wie die bei den Kindern, Danach fanden die Interventionen der Jugendlichen in einer ehemaligen Kaserne, im Münchner Hofgarten und am Münchner Flughafen statt.

Anhang 1 zu Kiesewetter J, Herbach N, Laudes I, Mayer J, Elgner V, Orle K, Grunow A, Langkau R, Gratzer C, Jansson AF. *Dog assisted education in children with rheumatic diseases and adolescents with chronic pain in Germany.* GMS J Med Educ. 2023;40(4):Doc44. DOI: 10.3205/zma001626

Supplement 1: Klinische Diagnosen der Teilnehmer

- Dateiname: diagnoses.xlsx

- Titel der Daten: Diagnose

- Beschreibung der Daten: Die Daten sind nach der Anzahl der Teilnehmer sortiert, die Diagnosen sind mit der entsprechenden ICD-10-Nummer eingetragen.

Die Kinder der Interventionsgruppe litten an rheumatischen Erkrankungen: juvenile idiopathische Arthritis (JIA, n=6), chronisch rezidivierende multifokale Osteomyelitis (CRMO, n=4), Arthritis (n=3) und Dermatomyositis (n=1). Die Kinder der Kontrollgruppe hatten JIA (11), CRMO (n=3) und Polyarthritis (n=2). Die Krankheiten der Kinder waren nicht unbedingt mit Schmerzen verbunden. Schmerzen beeinflussen die Lebensqualität, und chronische Schmerzsyndrome stellen eine Herausforderung in der Behandlung dar - nicht nur bei rheumatischen Erkrankungen. Daher wurden für die zweite Gruppe Jugendliche rekrutiert, die unter chronischen Schmerzen litten. Die Jugendlichen der Interventionsgruppe wiesen ein chronisches Schmerzsyndrom (n=7), Arthritis (n=1), JA (n=2) und Fibromyalgie (n=1), chronische Erkrankungen des Magen-Darm-Trakts (n=3) und Migräne (n=1) auf, und einige zeigten gleichzeitig Depressionen (n=2). Bei den Jugendlichen der Kontrollgruppe wurden ein chronisches Schmerzsyndrom (n=7), JA (n=3), chronische Gelenkschmerzen (n=1) und Weichteilrheuma (n=1) sowie eine juvenile Kyphose (n=1) und eine juvenile Osteoporose (n=1) diagnostiziert.

Supplement 2: Methoden für zusätzliche Fragebögen

Überblick: Der Deutsche Schmerzfragebogen für Kinder, Jugendliche und Eltern (DSF-KJ) wurde eingesetzt, um die Schmerzmerkmale der jugendlichen Teilnehmer zu analysieren. Die Visuelle Analogskala (VAS) ist ein Fragebogen zur Selbsteinschätzung von Schmerzen und wurde nur bei Kindern eingesetzt. Die Child Behaviour Check List (CBCL/4-18) wurde eingesetzt, um die jugendlichen Teilnehmer im Hinblick auf pathologische Werte für Kompetenzen (Aktivitäten, Soziales, Schule) und Syndrome (z. B. ängstlich/depressiv, somatische Beschwerden) zu und charakterisieren. Das Junior Temperament- und Charakterinventor (JTCI) wurde eingesetzt, um die Gesamtpersönlichkeit der Jugendlichen zu charakterisieren. Der Fragebogen zur Emotionsregulation bei Kindern und Jugendlichen (FEEL-KJ) diente dazu, Veränderungen in den adaptiven und maladaptiven Strategien der Jugendlichen im Zeitverlauf der Studie zu analysieren, die zu einer besseren Schmerzbewältigung führen könnten, die mit dem PPCI bewertet wurde. Der Fragebogen zu Stärken und Schwierigkeiten (SDO) und das Depressionsinventar für Kinder und Jugendliche (DIKJ) wurden verwendet, um psychiatrische Störungen bei Kindern zu ermitteln. Darüber hinaus wurde ein Fragebogen erstellt, um externe Einflüsse zu ermitteln, die sich auf das Ergebnis der Intervention auswirken könnten. Das Coping Health Inventory for Parents (CHIP) wurde von den Eltern der Jugendlichen ausgefüllt, um zu prüfen, ob sich die Schmerzbewältigung durch die Interventionen verbessert. Ein Fragebogen zur Bewertung des subjektiven Ergebnisses der Interventionen wurde auch von den Jugendlichen ausgefüllt.

DSF-KJ (Deutscher Schmerzfragebogen für Kinder, Jugendliche und Eltern)

- Dateiname: dsf_15-18.xlsx

- Titel der Daten: Fragebogendaten

- Beschreibung der Daten: Die Daten sind in vier Karten sortiert: Patienteninfo, Schmerzqualität, Schmerzintensität und negativer Einfluss des Schmerzes auf das tägliche Leben.

Zur Analyse der Schmerzeigenschaften wurde das DSF-KJ eingesetzt. Dieser Fragebogen ist ein modulares Selbsteinschätzungsinstrument zur Charakterisierung von Schmerzen. Der Basisfragebogen umfasst 57 Items zu folgenden Themen:

1. demografische Daten (sieben Items, z. B. Alter, Geschlecht, Schulform, Beruf der Eltern)
2. Schmerzcharakteristika (22 Items, Lokalisation, Häufigkeit, Dauer, zeitliche Veränderungen, Intensitätsskala - Numerische Rating-Skala (NRS) - von 0-10, Schmerzempfindungsskala (SES) und andere) [10]. Schmerzverursachende, schmerzlindernde und schmerzverstärkende Bedingungen (sechs Items)
3. medizinische Unterlagen (neun Items, z. B. frühere und aktuelle Behandlung und Medikation, Diagnose, Anzahl der Ärzte)
4. schmerzbedingte Behinderung (sieben Items, verpasste Aktivitäten und Schultage, Summe des Paediatric Pain Disability Index, PPDI [11]
5. kognitiv-emotionale und verhaltensbezogene Folgen und subjektives Krankheitskonzept (sechs Items, z. B. Reaktion der Jugendlichen und der Eltern auf die Schmerzen, Erwartungen in Bezug auf die Schmerzbewältigung und auf Schmerzfreiheit)

Der DSF-KJ wurde von den Jugendlichen vor und drei Monate nach Beginn der Intervention sowie eine Woche und sechs Monate nach Beendigung der Studie beantwortet (Tabelle 2).

Visuelle Analogskala (VAS)

- Dateiname: VAS.xlsx
- Titel der Daten: Fragebogendaten
- Beschreibung der Daten: Die Daten werden in einer Karteikarte mit der Patientenkennung, dem Zeitpunkt der Messung und der Schmerzhäufigkeit als abhängige Variable sortiert.

Die VAS ist ein Fragebogen zur Selbstbeurteilung von Schmerzen (Ja/Nein-Entscheidung), Schmerzhäufigkeit (dreistufige Ratingskala „selten", „oft", „ständig“) und Schmerzintensität (zehnstufige Ratingskala von „nicht vorhanden" bis „unerträglich“). Er wurde bei Kindern (7-12 Jahre) vor, sechs Monate nach Beginn und eine Woche nach Abschluss der Studie durchgeführt.

CBCL/4-18 (Child Behaviour Check List)

- Dateiname: cbcl_4-18.xlsx
- Titel der Daten: Fragebogendaten
- Beschreibung der Daten: Die Daten sind in zwei Karten sortiert: Kompetenzen, die die Fähigkeiten und ihre Werte im Vergleich zu den Mittelwerten zeigen, und Syndromskalen, die zeigen, wie die Krankheit das Leben der Betroffenen beeinflusst.

Der CBCL/4-18 (Achenbach 1991) ist ein validierter Fragebogen zur Bewertung der Eltern, der zur 2 Bestimmung der sozialen Kompetenzen und Syndrome verwendet wird. Er besteht aus zwei Teilen, wobei der erste die Kompetenzen anhand von drei Skalen (Aktivitäten, Soziales

und Schule) und Score sieben Items ermittelt. Der Gesamtkompetenzwert setzt sich aus der Summe der drei Skalenwerte zusammen. T-Scores unter 37 gelten als klinisch.

Im zweiten Teil werden Syndrom- und Problemskalen für Verhaltensauffälligkeiten, emotionale und somatische Auffälligkeiten ermittelt, wobei 113 Problem-Items für acht Problem-Skalen verwendet werden. Fünf der acht Skalen werden zu einem Maß für internalisierende und externalisierende Probleme zusammengefasst. Internalisierende Probleme bestehen aus drei Skalen: ängstlich/depressiv, zurückgezogen und somatische Beschwerden. Externalisierende Probleme umfassen die beiden Dimensionen kriminelles und aggressives Verhalten. Die anderen drei Skalen, die sich nicht in die beiden Untergruppen einordnen lassen, sind Sozial-, Denk- und Aufmerksamkeitsprobleme. Alle Items werden addiert, um die Gesamtproblemskala zu erstellen. Te Rohwerte werden T-Scores zugeordnet, und Syndrom-Scores über 70 und externalisierende, internalisierende sowie Gesamt-Scores über 63 gelten als klinisch.

Der CBCL/4-18-Test wurde von den Eltern der Jugendlichen eine Woche und sechs Monate nach Abschluss der Studie beantwortet.

JTCI 12-18R (Junior-Temperament und Charakterinventar)

- Dateiname: JTCI 12 18.xlsx

- Titel der Daten: Fragebogendaten-Scores

- Beschreibung der Daten: Die Daten sind in Karten für die Teil-Scores und die erste Karte als aggregierter Score für die Anzahl der Teilnehmer dargestellt.

Der JTCI 12-18R ist ein objektiver, zuverlässiger und validierter Fragebogen zur Selbsteinschätzung, der aus 103 Items besteht und zur Bestimmung der Gesamtpersönlichkeit eingesetzt wird (Goth and Schmeck 2009). Der JTCI 12-18R umfasst die vier

Temperamentsskalen Neuheitssuche, Schadensvermeidung, Belohnungsabhängigkeit und Beharrlichkeit sowie die Charakterskalen Selbststeuerung, Kooperationsbereitschaft und Selbsttranszendenz. Die Temperamentsskalen beschreiben Unterschiede in virtuellen automatischen emotionalen Reaktionen und geben Aufschluss über Persönlichkeitsstile. Die Charakterskalen erfassen Unterschiede in zentralen Selbstkonzepten und sind ein Indikator für die persönliche Reife. Temperament ist nach Cloninger die Grundlage, Charakter ist das, was man daraus macht. Beide zusammen tragen zur Persönlichkeit des Einzelnen bei.

Die Punktwerte der verschiedenen Items werden zu Rohwerten summiert und können zu T-Scores zusammengefasst werden. T-Scores unter 40 gelten als unterdurchschnittlich, und über 60 als überdurchschnittlich.

Der JTCI wurde bei Jugendlichen vor Beginn und eine Woche nach Beendigung der Studie eingesetzt.

FEEL-KJ (Fragebogen zur Emotionsregulation bei Kindern und Jugendlichen)

- Dateiname: Feel.xlsx
- Titel der Daten: Fragebogendaten
- Beschreibung der Daten: Die Daten sind in Karten für T-Scores und die erste Karte als aggregierter Score nach der Anzahl der Teilnehmer.

Der FEEL-KJ (Grob and Smolemski 2009) ist eine validierte Selbsteinschätzungsskala, mit der die emotionale Regulation von Angst, Traurigkeit und Aggression in Bezug auf das subjektive Wohlbefinden einer Person ermittelt werden kann. Dieser Test kann zur Bestimmung von Ressourcenprofilen, der Entwicklung von Emotionsregulationsstrategien bei Kindern, psychosozialen Kompetenzen, Stresswahrnehmung und -bewältigung sowie zur Messung von Fortschritten bei Interventionsprogrammen verwendet werden. Adaptive und maladaptive

Strategien werden durch Summieren der jeweiligen Skalen berechnet und T-Scores zugeordnet. T-Scores unter 40 (adaptiv) und über 60 (maladaptiv) bedeuten eine unzureichende Anwendung von Emotionsregulationsstrategien.

Der FEEL-Test wurde von den Jugendlichen vor und zwei Monate nach Beginn der AAE sowie eine Woche und sechs Monate nach Beendigung der Studie ausgefüllt.

SDQ (Fragebogen zu Stärken und Schwierigkeiten)

- Dateiname: SDQ.xlsx
- Titel der Daten: Fragebogendaten
- Beschreibung der Daten: Die Daten sind nach der Anzahl der Teilnehmer und der Gruppe, der sie angehören, sortiert, und der Wert sowie der aggregierte Score sind in derselben Karte enthalten.

Der SDO kann das Vorliegen einer psychiatrischen Störung mit guter Spezifität und mäßiger Sensitivität vorhersagen (sdqinfo.org). Der SDQ ist ein Fragebogen zur Fremdbeurteilung (Eltern oder Lehrer) oder zur Selbstbeurteilung (ab 11 Jahren), der 25 Items enthält, die sich aus fünf Skalen mit jeweils fünf Items zusammensetzen. Die dreistufige Bewertungsskala reicht von „trifft nicht zu“ über „trifft eher zu“ bis „trifft sicher zu“. Die Rohwerte für „trifft nicht zu und „trifft sicher zu" Scores variieren je nach Item (0 oder 2), „trifft eher zu™ wird immer mit 1 bewertet. Die Werte für die fünf Skalen reichen von 0 bis 10. Der Gesamtschwierigkeits-Score errechnet sich aus den vier Skalen und reicht folglich von 0 bis 40. Rohwerte über 16 gelten als abnormal (Goodman 1997, Woerner, Becker et al. 2002, Goodman, Ford et al. 2004). Der SDQ wurde von den Eltern der jüngeren Altersgruppe vor, sechs Monate nach Beginn und zwei Wochen nach Abschluss der Studie beantwortet.

Der DIKJH wurde eingesetzt, um Kinder mit schweren Depressionen auszuschließen. Der DIKJ (Naab, Hauer et al. 2015) ist ein zuverlässiges und validiertes Selbsteinschätzungsinstrument zur Untersuchung des Grades der Depression bei Kindern und Jugendlichen. Der DIKJ besteht aus 26 Items mit einer dreistufigen Skala: keine/selten, mäßig/gelegentlich und 'Schwer/meistens 0-3. Rohwerte über 17 sind auffällig für einen bestimmten Grad der Depression. Der DIKJ wurde in der jüngeren Altersgruppe vor Beginn der Studie, sechs Monate nach Beginn und zwei Wochen nach Abschluss der Studie eingesetzt.

CHIP-D (Coping Health Inventory für Eltern)

- Dateiname: Chip-D.xlsx
- Titel der Daten: Fragebogendaten
- Beschreibung der Daten: Die Daten sind nach der Anzahl der Teilnehmer auf zwei Karten sortiert. Die zweite Karte (Messwdh) zeigt die Daten der anderen Messzeitpunkte.

Der CHIP ist ein konsistentes, zuverlässiges und validiertes Selbsteinschätzungsinstrument zur Ermittlung der Fähigkeit von Eltern, mit einer chronischen Erkrankung ihrer Kinder umzugehen, und enthält 45 Items (MacCubbin 2001). Die vierstufige Ratingskala reicht von 0=nicht, 1=wenig, 2-mäßig bis 3=sehr hilfreich. Die Summe aller 45 Items ergibt die Gesamtskala (CHIP-ALL). Die drei wichtigsten Unterskalen (Dimensionen) sind

1. Aufrechterhaltung der familiären Integration, Kooperation und optimistische Sicht der Situation (19 Items, CHIP-FAM)
2. Aufrechterhaltung der sozialen Unterstützung, des Selbstwertgefühls und der psychologischen Stabilität (18 Items, CHIP-SUP)

3. Verständnis der Gesundheitssituation durch Kommunikation mit dem medizinischen Personal und anderen Eltern (acht Items, CHIP-MED). Die Rohwerte der Skala wurden in Prozentränge umgewandelt.

Der CHIP wurde vor und am Ende des Interventionsprogramms durchgeführt.

Externe Einflüsse

Externe Einflüsse auf das Interventionsprogramm wurden in der Gruppe der Jugendlichen berücksichtigt.

Eine Woche und sechs Monate nach Abschluss der Studie wurden die Jugendlichen nach unterstützenden Instrumenten und positiven oder negativen Veränderungen in ihrem Leben befragt.

Eine Woche nach Abschluss profitierten drei Jugendliche aus der Interventions- und fünf aus der Kontrollgruppe von Physiotherapie, Gymnastik, Sport, Heilpraktikertherapie und Psychotherapie. Fünf Kinder aus der Interventionsgruppe und drei aus der Kontrollgruppe fanden Unterstützung durch neue Freunde. Negative Ereignisse waren der Tod von nahestehenden Personen (1 Interventions-, 1 Kontrollgruppe) und die Trennung von den Eltern (1 Kontrollgruppe) oder vom Freund (1 Interventionsgruppe). Drei Teilnehmer aus der Interventionsgruppe berichteten über Probleme in der Schule.

Sechs Monate nach Abschluss des AAP berichteten vier Jugendliche über positive Auswirkungen von Physiotherapie und Osteopathie auf ihre Krankheit. Positive Ereignisse wurden von acht Jugendlichen aus der Interventionsgruppe und sechs aus der Kontrollgruppe genannt, z. B. Schulabschluss, Arbeit oder Beginn einer Ausbildung und neue Freunde. Keines der Mädchen erwähnte Probleme in der Schule oder bei der Arbeit.

Bewertung des Interventionsprogramms

Zur Ermittlung der Bewertung und des subjektiven Ergebnisses der Interventionen wurde ein Fragebogen von der eo ipso Strategie & Entwicklung GmbH (www.eo-ipso.com) entwickelt. Der eo ipso Fragebogen ist ein Fragebogen mit Freitext zur Selbst- und Elterneinschätzung des Interventionsprogramms. Ermittelt wurden die Gesamtbewertung (positiv, negativ) und die Auswirkungen auf die Psyche bzw. die körperliche Konstitution sowie der Einfluss auf das Schmerzempfinden der Heranwachsenden. Die Eltern wurden gebeten, Veränderungen des Kindes während des Interventionsprogramms zu beschreiben, die sie als Ergebnis der Intervention diese hatten ansehen, insbesondere wenn diese einen Einfluss auf die Schmerzen hatten. Der eo ipso-Fragebogen wurde von den Jugendlichen nach der letzten Intervention ausgefüllt.

Supplement 3: Detaillierte statistische Analysen der wichtigsten Parameter

Abkürzungen

T: Zeitpunkt

TO: vor Beginn des Interventionsprogramms

TI-TX: ein bis & Monate nach Beginn des Programms

T final: letzte Intervention

TOM: sechs Monate nach der Intervention

PedsQl

- Dateiname: PedsQL.xlsx

- Titel der Daten: Fragebogendaten

- Beschreibung der Daten: Die Daten sind nach der Anzahl der Teilnehmer auf zwei Karten sortiert. Die letzte Spalte stellt den aggregierten Score dar.

Kinder (Selbsteinschätzung)

Deskriptive Statistik				
	Gruppe	Mittel	SD	N
SMITTEL (PedsQL_C_t1)	Intervention	76.9179	16.03057	14
	Kontrolle	86.4475	9.16035	16
	Gesamt	82.0003	13.49008	30
SMITTE L(PedsQL_C_t2)	Intervention	85.9818	11.85440	14
	Kontrolle	90.7871	6.28607	16
	Gesamt	88.5446	9.45403	30
SMITTEL (PedsQL_C_t3)	Intervention	86.5714	11.22074	14
	Kontrolle	91.4418	6.83786	16
	Gesamt	89.1689	9.31300	30
SMITTEL (PedsQL_C_t4)	Intervention	88.8634	8.77118	14
	Kontrolle	92.5275	6.72325	16
	Gesamt	90.8176	7.83100	30
SMITTEL (PedsQL_C_t5)	Intervention	87.4348	8.90996	14
	Kontrolle	93.4044	6.72474	16
	Gesamt	90.6186	8.25551	30
SMITTEL (PedsQL_C_t6)	Intervention	92.4990	5.70701	14
	Kontrolle	91.7629	8.27568	16
	Gesamt	92.1064	7.08266	30
SMITTEL (PedsQL_C_t7)	Intervention	90.2958	9.46693	14
	Kontrolle	93.0919	6.58804	16
	Gesamt	91.7870	8.03978	30

SMITTEL (PedsQL_C_t8)	Intervention	89.8612	8.75641	14
	Kontrolle	91.9838	7.98614	16
	Gesamt	90.9932	8.27770	30
SMITTEL (PedsQL_C_t9)	Intervention	93.4293	4.81754	14
	Kontrolle	92.4481	7.86465	16
	Gesamt	92.9060	6.53028	30
SMITTEL (PedsQL_C_t10)	Intervention	90.0185	11.79506	14
	Kontrolle	93.7519	5.31697	16
	Gesamt	92.0097	8.97646	30
SMITTEL (PedsQL_C_t11)	Intervention	90.7993	10.15334	14
	Kontrolle	93.8856	6.74571	16
	Gesamt	92.4453	8.49719	30
SMITTEL (PedsQL_C_t12)	Intervention	89.0340	10.14511	14
	Kontrolle	93.2750	7.51237	16
	Gesamt	91.2959	8.94203	30

Kinder ANOVA mit wiederholten Messungen und der Gruppe als Variable zwischen den Teilnehmern

Quelle		df	F	Sig.	Partielles Eta-Quadrat	Beobachtete Aussagekraft (Observed Power)[a]
PedsQL_C	Greenhouse-Geisser	5.373	8.173	.000	.226	1.000
PedsQL_C * Gruppe	Greenhouse-Geisser	5.373	1.784	.114	.060	.623
Fehler (PedsQL_C)	Greenhouse-Geisser	150.456				

Kinder-Eltern Beurteilung

Deskriptive Statistik				
	Gruppe	Mittel	SD	N
SMITTEL (PedsQL_C_P_t1)	Intervention	77.8300	12.38876	14
	Kontrolle	83.1531	12.05677	16
	Gesamt	80.6690	12.29987	30
SMITTEL (PedsQL_C_P_t2)	Intervention	81.9396	11.63843	14
	Kontrolle	86.0170	8.38422	16
	Gesamt	84.1142	10.06780	30
SMITTEL (PedsQL_C_P_t3)	Intervention	81.0462	13.71659	14
	Kontrolle	86.7783	8.69802	16
	Gesamt	84.1033	11.48620	30
SMITTEL (PedsQL_C_P_t4)	Intervention	82.7265	10.85143	14
	Kontrolle	91.3400	7.97397	16
	Gesamt	87.3203	10.23606	30
SMITTEL (PedsQL_C_P_t5)	Intervention	85.0463	10.17073	14
	Kontrolle	89.4019	7.85419	16
	Gesamt	87.3693	9.11941	30
SMITTEL (PedsQL_C_P_t6)	Intervention	85.1590	8.13858	14
	Kontrolle	88.2463	10.89973	16
	Gesamt	86.8055	9.67453	30
SMITTEL (PedsQL_C_P_t7)	Intervention	85.3529	10.70834	14
	Kontrolle	86.5481	11.15561	16
	Gesamt	85.9904	10.77685	30
SMITTEL (PedsQL_C_P_t8)	Intervention	85.5398	10.71594	14
	Kontrolle	83.8594	11.21498	16
	Gesamt	84.6436	10.82865	30
SMITTEL (PedsQL_C_P_t9)	Intervention	89.3637	5.12947	14
	Kontrolle	86.4813	9.94524	16
	Gesamt	87.8264	8.06804	30
SMITTEL (PedsQL_C_P_t10)	Intervention	85.0936	10.75530	14
	Kontrolle	88.0194	9.98627	16
	Gesamt	86.6540	10.27819	30
SMITTEL (PedsQL_C_P_t11)	Intervention	86.8000	8.64195	14
	Kontrolle	88.6556	9.60704	16
	Gesamt	87.7897	9.06114	30
SMITTEL (PedsQL_C_P_t12)	Intervention	85.4443	11.49208	14
	Kontrolle	85.3938	11.18336	16
	Gesamt	85.4173	11.13075	30

Bewertung der Kinder durch die Eltern ANOVA mit wiederholten Messungen und der Gruppe als Variable zwischen den Teilnehmern

Quelle		df	F	Sig.	Partielles Eta-Quadrat	Beobachtete Aussagekraft (Observed Power)[a]
PedsQL_C_P	Greenhouse-Geisser	6.671	3.197	.004	.102	.939
PedsQL_C_P * Gruppe	Greenhouse-Geisser	6.671	1.894	.076	.063	.727
Fehler (PedsQL_C_P)	Greenhouse-Geisser	186.788				

PPCI

- Dateiname: PPCI.xlsx

- Titel der Daten: Fragebogendaten

- Beschreibung der Daten: Die Daten sind nach der Anzahl der Teilnehmer und Gruppe auf jeder Karte sortiert. Die erste Karte steht für die Schmerzbewältigung, die zweite für die soziale Unterstützung, die dritte für die positive Selbstinstruktion und die vierte für das Datum der Datenerfassung.

Kinder

Deskriptive Statistik				
	Gruppe	Mittel	SD	N
PPCI T0	Intervention	26.7692	28.58074	13
	Kontrolle	22.2000	7.38918	15
	Gesamt	24.3214	19.91845	28
PPCI T_final	Intervention	19.8462	6.26958	13
	Kontrolle	22.4667	8.30548	15
	Gesamt	21.2500	7.41682	28

Quelle		df	F	Sig.	Partielles Eta-Quadrat	Beobachtete Aussagekraft (Observed Power)[a]
PPCI	Sphärizitätsannahme	1	.647	.429	.024	.121
PPCI * Gruppe	Sphärizitätsannahme	1	.755	.393	.028	.133
Fehler (PPCI)	Sphärizitätsannahme	26				

Jugendliche

Deskriptive Statistik

	Gruppe	Mittel	SD	N
Gesamt PPCI T1	Intervention	2.9192	.53566	12
	Kontrolle	2.6850	.74112	12
	Gesamt	2.8021	.64360	24
Gesamt PPCI T_final	Intervention	2.6908	.76382	12
	Kontrolle	2.2917	.65217	12
	Gesamt	2.4913	.72389	24
Gesamt PPCI T6M follow-up	Intervention	2.7242	.69378	12
	Kontrolle	2.5508	.72818	12
	Gesamt	2.6375	.70117	24

Quelle		df	F	Sig.	Partielles Eta-Quadrat	Beobachtete Aussagekraft (Observed Power)[a]
Gesamt	Sphärizitätsannahme	2	2.517	.092	.103	.478
Gesamt * Gruppe	Sphärizitätsannahme	2	.355	.703	.016	.103
Fehler (Gesamt)	Sphärizitätsannahme	44				

Jugendliche (Selbsteinschätzung)

Deskriptive Statistik				
	Gruppe	Mittel	SD	N
Gesamt Score T1	Intervention	63.6000	15.01999	10
	Kontrolle	58.0000	20.21700	12
	Gesamt	60.5455	17.85851	22
Gesamt Score T2	Intervention	65.7000	15.42040	10
	Kontrolle	61.2500	19.93227	12
	Gesamt	63.2727	17.75275	22
Gesamt Score T3	Intervention	69.6000	16.90628	10
	Kontrolle	63.4167	17.33341	12
	Gesamt	66.2273	17.02360	22
Gesamt Score T4	Intervention	63.3000	16.65366	10
	Kontrolle	67.6667	21.01659	12
	Gesamt	65.6818	18.84621	22
Gesamt Score T5	Intervention	64.8000	13.79855	10
	Kontrolle	67.2500	19.67982	12
	Gesamt	66.1364	16.91237	22
Gesamt Score T6	Intervention	65.2000	15.38975	10
	Kontrolle	61.1667	20.45764	12
	Gesamt	63.0000	18.02644	22
Gesamt Score T6M follow-up	Intervention	67.9000	17.54011	10
	Kontrolle	77.4167	12.07144	12
	Gesamt	73.0909	15.22188	22

Jugendliche ANOVA mit wiederholten Messungen und der Gruppe als Variable zwischen den Teilnehmern

Quelle		df	F	Sig.	Partielles Eta-Quadrat	Beobachtete Aussagekraft (Observed Power)[a]
Gesamt	Greenhouse-Geisser	2.873	2.013	.125	.091	.480
Gesamt * Gruppe	Greenhouse-Geisser	2.873	1.312	.280	.062	.325
Fehler (Gesamt)	Greenhouse-Geisser	57.466				

Jugendliche-Eltern-Beurteilung

Deskriptive Statistik				
	Gruppe	Mittel	SD	N
Gesamt Score T1	Eltern Intervention	67.8333	13.61706	12
	Eltern Kontrolle	55.2308	21.85236	13
	Gesamt	61.2800	19.10611	25
Gesamt Score T2	Eltern Intervention	67.9167	12.83786	12
	Eltern Kontrolle	58.1538	26.44758	13
	Gesamt	62.8400	21.21454	25
Gesamt Score T3	Eltern Intervention	72.0000	12.14309	12
	Eltern Kontrolle	57.2308	22.28659	13
	Gesamt	64.3200	19.30397	25
Gesamt Score T4	Eltern Intervention	66.9167	21.72329	12
	Eltern Kontrolle	65.6923	21.67712	13
	Gesamt	66.2800	21.25151	25
Gesamt Score T 5	Eltern Intervention	73.6667	13.60036	12
	Eltern Kontrolle	61.9231	20.97801	13
	Gesamt	67.5600	18.45734	25
Gesamt Score T6	Eltern Intervention	69.6667	17.80364	12
	Eltern Kontrolle	64.0769	20.38570	13
	Gesamt	66.7600	19.00500	25

Bewertung der Jugendlichen durch die Eltern ANOVA mit wiederholten Messungen und der Gruppe als Variable zwischen den Teilnehmern

Quelle		df	F	Sig.	Partielles Eta-Quadrat	Beobachtete Aussagekraft (Observed Power)[a]
Gesamt_Eltern	Sphärizitätsannahme	5	1.180	.323	.049	.407
Gesamt_Eltern * Gruppe	Sphärizitätsannahme	5	1.279	.278	.053	.440
Fehler (Gesamt_Eltern)	Sphärizitätsannahme	115				

STAIC-S (Kinder)

- Dateiname: STAIState.xlsx

- Titel der Daten: Fragebogendaten

- Beschreibung der Daten: Die Daten sind nach der Anzahl der Teilnehmer und der Gruppe sortiert. Die Daten werden für jeden Messzeitpunkt dargestellt.

Deskriptive Statistik				
	Vorher-Nachher	Mittel	SD	N
SMITTEL (STAIC_S_t1_v)	Vorher	29.3077	5.63485	14
	Nachher	27.2308	4.38799	14
	Gesamt	28.2692	5.06722	28
SMITTEL (STAIC_S_t2_v)	Vorher	26.3636	2.83583	14
	Nachher	26.5385	3.81526	14
	Gesamt	26.4510	3.29978	28
SMITTEL (STAIC_S_t3_v)	Vorher	25.3077	2.69999	14
	Nachher	24.5385	3.24903	14
	Gesamt	24.9231	2.95736	28
SMITTEL (STAIC_S_t4_v)	Vorher	26.0000	4.18789	14
	Nachher	24.3846	3.85384	14
	Gesamt	25.1923	4.03385	28
SMITTEL (STAIC_S_t5_v)	Vorher	26.5000	4.64923	14
	Nachher	25.4167	3.77152	14
	Gesamt	25.9583	4.19052	28
SMITTEL (STAIC_S_t6_v)	Vorher	25.8462	4.25795	14
	Nachher	24.7692	3.76530	14
	Gesamt	25.3077	3.98199	28
SMITTEL (STAIC_S_t7_v)	Vorher	26.8182	3.13340	14
	Nachher	27.5455	4.54657	14
	Gesamt	27.1818	3.84932	28
SMITTEL (STAIC_S_t8_v)	Vorher	25.5000	3.15009	14
	Nachher	25.4167	3.81209	14
	Gesamt	25.4583	3.43169	28

SMITTEL (STAIC_S_t9_v)	Vorher	26.1111	4.32247	14
	Nachher	25.1111	2.33516	14
	Gesamt	25.6111	3.44683	28
SMITTEL (STAIC_S_t10_v)	Vorher	26.5000	5.18504	14
	Nachher	25.9286	4.51432	14
	Gesamt	26.2143	4.77925	28
SMITTEL (STAIC_S_t11_v)	Vorher	26.6429	5.85212	14
	Nachher	25.8571	4.81755	14
	Gesamt	26.2500	5.27485	28
SMITTEL (STAIC_S_t12_v)	Vorher	25.5000	4.30116	14
	Nachher	23.5000	3.14398	14
	Gesamt	24.5000	3.83454	28

Quelle		df	F	Sig.	Partielles Eta-Quadrat	Beobachtete Aussagekraft (Observed Power)[a]
STAIC	Greenhouse-Geisser	5.455	3.018	.010	.104	.876
STAIC * Pre_Post	Greenhouse-Geisser	5.455	.496	.794	.019	.188
Fehler (STAIC)	Greenhouse-Geisser	141.839				

STAI-S (Jugendliche)

Deskriptive Statistik				
	Vorher Nachher	Mittel	SD	N
T1	Vorher	40.75	9.401	12
	Nachher	28.92	3.753	12
	Gesamt	34.83	9.249	24
T2	Vorher	36.33	4.334	12
	Nachher	28.50	6.360	12
	Gesamt	32.42	6.659	24
T3	Vorher	39.25	7.187	12
	Nachher	31.92	4.400	12
	Gesamt	35.58	6.928	24
T4	Vorher	37.83	8.222	12
	Nachher	31.92	3.343	12
	Gesamt	34.88	6.842	24
T5	Vorher	42.75	7.313	12
	Nachher	29.83	5.289	12
	Gesamt	36.29	9.082	24
T6	Vorher	44.08	8.393	12
	Nachher	31.67	6.415	12
	Gesamt	37.88	9.674	24

Quelle		df	F	Sig.	Partielles Eta-Quadrat	Beobachtete Aussagekraft (Observed Power)[a]
STAI	Greenhouse-Geisser	2.926	2.283	.089	.094	.544
STAI * Vorher_Nachher	Greenhouse-Geisser	2.926	1.589	.202	.067	.394
Fehler (STAI)	Greenhouse-Geisser	64.379				

Statistik für gepaarte Stichproben					
		Mittel	N	SD	Standard Fehler Mittel
Paar 1	T1 Vorher	40.75	12	9.401	2.714
	T1 Nachher	28.92	12	3.753	1.083
Paar 2	T2 Vorher	36.33	12	4.334	1.251
	T2 Nachher	28.50	12	6.360	1.836
Paar 3	T3 Vorher	39.25	12	7.187	2.075
	T3 Nachher	31.92	12	4.400	1.270
Paar 4	T4 Vorher	37.83	12	8.222	2.374
	T4 Nachher	31.92	12	3.343	.965
Paar 5	T5 Vorher	42.75	12	7.313	2.111
	T5 Nachher	29.83	12	5.289	1.527
Paar 6	T6 Vorher	44.08	12	8.393	2.423
	T6 Nachher	31.67	12	6.415	1.852

Korrelationen für gepaarte Stichproben				
		N	Korrelation	Signifikanz
Paar 1	T1 Vorher - Nachher	12	.056	.863
Paar 2	T2 Vorher - Nachher	12	.475	.119
Paar 3	T3 Vorher - Nachher	12	.297	.349
Paar 4	T4 Vorher - Nachher	12	-.477	.117
Paar 5	T5 Vorher - Nachher	12	-.043	.893
Paar 6	T6 Vorher - Nachher	12	.441	.151

Test gepaarte Stichproben									
		Gepaarte Differenzen					T	df	Sig. (2-armig)
		Mittel	SD	Standard Fehler Mittel	95% Konfidenzintervall für die Differenz: Oberes	95% Konfidenzintervall für die Differenz: Unteres			
Paar 1	T1 Vorher - Nachher	11.833	9.925	2.865	5.527	18.140	4.130	11	.002
Paar 2	T2 Vorher - Nachher	7.833	5.750	1.660	4.180	11.487	4.719	11	.001
Paar 3	T3 Vorher - Nachher	7.333	7.228	2.087	2.741	11.926	3.515	11	.005
Paar 4	T4 Vorher - Nachher	5.917	10.247	2.958	-.594	12.427	2.000	11	.071
Paar 5	T5 Vorher - Nachher	12.917	9.209	2.658	7.065	18.768	4.859	11	.001
Paar 6	T6 Vorher - Nachher	12.417	8.005	2.311	7.330	17.503	5.373	11	.000

Deskriptive Statistik			
	Mittel	SD	N
T1 Vorher	40.75	9.401	12
T2 Vorher	36.33	4.334	12
T3 Vorher	39.25	7.187	12
T4 Vorher	37.83	8.222	12
T5 Vorher	42.75	7.313	12
T6 Vorher	44.08	8.393	12

Quelle		df	F	Sig.	Partielles Eta-Quadrat	Beobachtete Aussagekraft (Observed Power)[a]
Vor_SMEAM	Greenhouse-Geisser	2.575	2.025	.140	.155	.432
Fehler (Vor_SMEAM)	Greenhouse-Geisser	28.321				

Deskriptive Statistik			
	Mittel	SD	N
T1 Nachher	28.92	3.753	12
T2 Nachher	28.50	6.360	12
T3 Nachher	31.92	4.400	12
T4 Nachher	31.92	3.343	12
T5 Nachher	29.83	5.289	12
T6 Nachher	31.67	6.415	12

Quelle		df	F	Sig.	Partielles Eta-Quadrat	Beobachtete Aussagekraft (Observed Power)[a]
Nach_SMEAM	Sphärizitätsannahme	5	1.676	.156	.132	.539
Fehler (Nach_SMEAM)	Sphärizitätsannahme	55				

Deskriptive Statistik			
	Mittel	SD	N
Differenz T1	11.833	9.9255	12
Differenz T2	7.833	5.7498	12
Differenz T3	7.333	7.2279	12
Differenz T4	5.917	10.2466	12
Differenz T5	12.917	9.2093	12
Differenz T6	12.417	8.0052	12

Quelle		df	F	Sig.	Partielles Eta-Quadrat	Beobachtete Aussagekraft (Observed Power)[a]
Diff_SMEAM	Sphärizitätsannahme	5	2.062	.084	.158	.642
Fehler (Diff_SMEAM)	Sphärizitätsannahme	55				

STAIC-T Angstmodell

- Dateiname: STAITrait.xlsx

- Titel der Daten: Fragebogendaten

- Beschreibung der Daten: Die Daten sind nach der Anzahl der Teilnehmer und der Gruppe sortiert. Die Daten werden für die beiden Messzeitpunkte dargestellt.

Deskriptive Statistik

	Gruppe	Mittel	SD	N
STAIC_T_T0	Intervention	32.00	7.328	14
	Kontrolle	29.88	7.070	16
	Gesamt	30.87	7.147	30
STAIC_T_T13	Intervention	30.43	6.465	14
	Kontrolle	26.56	5.597	16
	Gesamt	28.37	6.228	30

Quelle		df	F	Sig.	Partielles Eta-Quadrat	Beobachtete Aussagekraft (Observed Power)[a]
STAIC_T	Sphärizitätsannahme	1	3.442	.074	.109	.433
STAIC_T * Gruppe	Sphärizitätsannahme	1	.437	.514	.015	.098
Fehler (STAIC_T)	Sphärizitätsannahme	28				

Supplement 4: Ergebnisse der zusätzlichen Fragebögen

Zusammenfassung der Ergebnisse der zusätzlichen Fragebögen:

Kurz gesagt verringerten sich die externalisierenden und internalisierenden Scores sowie die Gesamt-Scores der Child Behaviour Check List (CBCL/4-18) innerhalb des Untersuchungszeitraums. In der Interventionsgruppe verringerten sich Externalisierungs- und Gesamt-Scores bis zum Ende der Studie signifikant. In der Kontrollgruppe waren die Werte für externalisierendes und internalisierendes Verhalten bei der Nachuntersuchung nach 6 Monaten signifikant niedriger. Über alle Teilnehmer und Zeitpunkte hinweg waren die Häufigkeiten der klinischen Werte für somatische Beschwerden (49 %) und internalisierendes Verhalten (32 %) hoch. Die Scores für Rückzug (23 %), die Gesamtskala des Syndroms (18 %) und ängstlich/depressiv (16 %) waren weniger häufig klinisch. Klinische Werte für delinquentes, externalisierendes, aggressives Verhalten sowie Denk- und Aufmerksamkeitsprobleme wurden selten festgestellt (1-7 %).

Die Werte des Junior Temperament- und Charakterinventars änderten sich während des Studienzeitraums nicht, und es gab keine Unterschiede zwischen den Gruppen. Allerdings zeigten etwa 40 % der Teilnehmer eine verminderte Selbststeuerung (T-Werte < 40), was auf eine verminderte persönliche Reife der an der Studie teilnehmenden Jugendlichen mit chronischen Schmerzen hindeutet. Eine verminderte Selbststeuerung ist typisch für einen .„unsicher-ineffektiven“ Stil, der z. B. durch Hilflosigkeit, Unzufriedenheit und Ziellosigkeit gekennzeichnet ist.

Nahezu die Hälfte der Jugendlichen Her Interventions- und Kontrollgruppe zeigte auffällig veränderte Temperamentsskalen, d. h. eine verringerte Neuheitssuche, die für einen „stoisch-dispassionierten“ Persönlichkeitsstil charakteristisch ist, und eine erhöhte Schadensvermeidung, die für einen „vorsichtig-ängstlichen" Persönlichkeitsstil (z. B. pessimistisch, zweifelnd, unsicher, schwach, schüchtern) typisch ist. Die Kombination aus geringer

Neuheitssuche und hoher Schadensvermeidung charakterisiert den Temperamentstyp zweiter Ordnung „starr-introvertiert*, der bei sieben Jugendlichen vor Beginn und sechs Jugendlichen nach Abschluss der Studie zu beobachten war.

Der Fragebogen zur Emotionsregulation bei Kindern und Jugendlichen (FEEL) hat sich im Untersuchungszeitraum nicht verändert, und es gab keine Unterschiede zwischen den Gruppen: Etwa 40 % der Jugendlichen mit chronischen Schmerzen verwenden seltener als die Normstichprobe adaptive Emotionsregulationsstrategien zur Förderung des Wohlbefindens (T-Scores für adaptive Strategien insgesamt unter 40), über 50 % zeigen unterdurchschnittliche Werte für die Skalen Ablenkung, Problemlösung und Akzeptanz. Etwa 50 % der Jugendlichen, die an dieser Studie teilnahmen, verwendeten Emotionsregulationsstrategien, die dem Wohlbefinden abträglich, sind (Gesamt-T-Scores für maladaptive Regulation über 60). Am bemerkenswertesten sind die überdurchschnittlich hohen Werte für sozialen Rückzug und Grübeln.

CBCL/4-18 (Child Behaviour Check List)

- Dateiname: cbcl_4-18.xISx

- Titel der Daten: Fragebogendaten

- Beschreibung der Daten: Die Daten sind in zwei Karten sortiert: Kompetenzen, die die Kompetenzen und ihre Werte im Vergleich zu den Mittelwerten zeigen, und Syndromskalen, die zeigen, wie die Krankheit das Leben der Betroffenen beeinflusst.

Der CBCL/4-18 wurde von den Eltern der Jugendgruppe eine Woche nach Abschluss der Intervention und bei der Nachuntersuchung nach 26 Wochen ausgefüllt.

Es gab keine signifikanten Veränderungen der verschiedenen Kompetenzwerte im Zeitverlauf, aber die Interaktion Zeit und Gruppe des Gesamtkompetenz-Scores war grenzwertig signifikant (p=0.054). Die Gesamtkompetenzwerte der Interventionsgruppe sanken leicht und die der Kontrollgruppe stiegen tendenziell vom Ende der Studie bis zur 26-wöchigen Nachbeobachtung. Die Kompetenzwerte der Interventions- und der Kontrollgruppe unterschieden sich nicht.

Die Internalisierungs-, Externalisierungs- und Gesamt-Scores der Syndromskalen veränderten sich mit der Zeit (p=0.02, F(,)= eta2-0,222), aber die Interaktion von Zeit und Gruppe war nicht signifikant. Über alle Teilnehmer hinweg waren die drei Haupt-Scores nach 26 Wochen Nachbeobachtung niedriger als eine Woche nach der Studie.

Deskriptive Statistik				
	Gruppe	Mittel	SD	N
Gesamt Kompetenz-Score T0	Intervention	61.57	11.984	7
	Kontrolle	52.50	16.887	10
	Gesamt	56.24	15.344	17
Gesamt Kompetenz-Score T_final	Intervention	56.14	12.890	7
	Kontrolle	51.20	11.163	10
	Gesamt	53.24	11.777	17
Gesamt Kompetenz-Score T6M follow-up	Intervention	53.43	14.293	7
	Kontrolle	57.10	12.206	10
	Gesamt	55.59	12.802	17

Quelle		df	F	Sig.	Partielles Eta-Quadrat	Beobachtete Aussagekraft (Observed Power)[a]
Gesamt	Greenhouse-Geisser	1.399	1.006	.356	.063	.178
Gesamt * Gruppe	Greenhouse-Geisser	1.399	3.754	.054	.200	.530
Fehler (Gesamt)	Greenhouse-Geisser	20.986				

Deskriptive Statistik				
	Gruppe	Mittel	SD	N
Gesamt Syndrom-Score T_final	Intervention	63.83	8.526	12
	Kontrolle	58.92	11.285	12
	Gesamt	61.38	10.099	24
Gesamt Syndrom-Score T6M Follow-up	Intervention	59.92	9.624	12
	Kontrolle	55.17	14.038	12
	Gesamt	57.54	12.018	24

Quelle		df	F	Sig.	Partielles Eta-Quadrat	Beobachtete Aussagekraft (Observed Power)[a]
Gesamt Syndrom-Score	Sphärizität sannahme	1	6.281	.020	.222	.669
Gesamt Syndrom * Gruppe	Sphärizität sannahme	1	.003	.957	.000	.050
Fehler (Gesamt Syndrom-Score)	Sphärizität sannahme	22				

JTCI (Junior- Temperament und Charakterinventar)

- Dateiname: JICL_12_18.XIsx-

- Titel der Daten: Fragebogendaten

- Beschreibung der Daten: Die Daten sind in Karten für die Teilergebnisse und die erste Karte als aggregierter Score für die Anzahl der Teilnehmer dargestellt.

Der JICI wurde von den Jugendlichen vor und eine Woche nach Abschluss des Programms ausgefüllt. Das Interventionsprogramm hatte keinen Einfluss auf das Temperament und die Charaktereigenschaften der Jugendlichen. Die ANOVA mit wiederholten Messungen ergab keine signifikanten Veränderungen in den sieben Skalen beider Gruppen im Zeitverlauf.

Anhang 1 zu Kiesewetter J, Herbach N, Laudes I, Mayer J, Elgner V, Orle K, Grunow A, Langkau R, Gratzer C, Jansson AF. *Dog assisted education in children with rheumatic diseases and adolescents with chronic pain in Germany.* GMS J Med Educ. 2023;40(4):Doc44. DOI: 10.3205/zma001626

Deskriptive Statistik				
	Gruppe	Mittel	SD	N
Neuheitssuche T0	Intervention	40.25	8.137	12
	Kontrolle	40.31	9.340	13
	Gesamt	40.28	8.600	25
Neuheitssuche T_final	Intervention	41.33	8.489	12
	Kontrolle	54.23	15.611	13
	Gesamt	48.04	14.076	25

Test der Innersubjektkontraste						
Quelle		df	F	Sig.	Partielles Eta-Quadrat	Beobachtete Aussagekraft (Observed Power)[a]
Neuheitssuche	Greenhouse-Geisser	1.000	5.900	.023	.204	.643
Neuheitssuche * Gruppe	Greenhouse-Geisser	1.000	4.319	.049	.158	.512
Fehler (Neuheitssuche)	Greenhouse-Geisser	23.000				

Deskriptive Statistik				
	Gruppe	Mittel	SD	N
Neuheitssuche T0	Intervention	59.75	9.097	12
	Kontrolle	53.38	16.251	13
	Gesamt	56.44	13.435	25
Neuheitssuche T_final	Intervention	59.67	10.688	12
	Kontrolle	47.54	16.616	13
	Gesamt	53.36	15.121	25

Test der Innersubjektkontraste						
Quelle		df	F	Sig.	Partielles Eta-Quadrat	Beobachtete Aussagekraft (Observed Power)[a]
Neuheitssuche	Greenhouse-Geisser	1.000	.606	.444	.026	.116
Neuheitssuche * Gruppe	Greenhouse-Geisser	1.000	.573	.457	.024	.112
Fehler (Neuheitssuche)	Greenhouse-Geisser	23.000				

Deskriptive Statistik				
	Gruppe	Mittel	SD	N
Beharrlichkeit T0	Intervention	52.25	7.375	12
	Kontrolle	45.62	15.735	13
	Gesamt	48.80	12.656	25
Beharrlichkeit T_final	Intervention	52.17	8.451	12
	Kontrolle	47.23	9.418	13
	Gesamt	49.60	9.133	25

Test der Innersubjektkontraste						
Quelle		df	F	Sig.	Partielles Eta-Quadrat	Beobachtete Aussagekraft (Observed Power)[a]
Beharrlichkeit	Greenhouse-Geisser	1.000	.055	.817	.002	.056
Beharrlichkeit * Gruppe	Greenhouse-Geisser	1.000	.068	.797	.003	.057
Fehler (Beharrlichkeit)	Greenhouse-Geisser	23.000				

Deskriptive Statistik				
	Gruppe	Mittel	SD	N
Kooperations-bereitschaft T0	Intervention	57.33	6.184	12
	Kontrolle	55.77	10.910	13
	Gesamt	56.52	8.813	25
Kooperations-bereitschaft T_final	Kooperations-bereitschaft	56.00	8.135	12
	Kontrolle	48.23	15.178	13
	Gesamt	51.96	12.697	25

Test der Innersubjektkontraste						
Quelle		df	F	Sig.	Partielles Eta-Quadrat	Beobachtete Aussagekraft (Observed Power)[a]
Kooperations-bereitschaft	Greenhouse-Geisser	1.000	1.776	.196	.072	.248
Kooperations-bereitschaft * Gruppe	Greenhouse-Geisser	1.000	.869	.361	.036	.145
Fehler (Kooperations-bereitschaft)	Greenhouse-Geisser	23.000				

Deskriptive Statistik				
	Gruppe	Mittel	SD	N
Selbststeuerung T0	Intervention	41.42	11.501	12
	Kontrolle	43.62	14.086	13
	Gesamt	42.56	12.692	25
Selbststeuerung T_final	Intervention	40.00	13.287	12
	Kontrolle	48.62	10.071	13
	Gesamt	44.48	12.285	25

Test der Innersubjektkontraste						
Quelle		df	F	Sig.	Partielles Eta-Quadrat	Beobachtete Aussagekraft (Observed Power)[a]
Selbststeuerung	Sphärizitätsannahme	1	.392	.537	.017	.092
Selbststeuerung * Gruppe	Sphärizitätsannahme	1	1.258	.274	.052	.189
Fehler (Selbststeuerung)	Sphärizitätsannahme	23				

Deskriptive Statistik				
	Gruppe	Mittel	SD	N
Selbsttrans-zendenz T0	Intervention	54.00	11.290	12
	Kontrolle	47.77	16.037	13
	Gesamt	50.76	14.039	25
Selbsttrans-zendenz T_final	Intervention	50.67	10.120	12
	Kontrolle	58.38	12.339	13
	Gesamt	54.68	11.771	25

Test der Innersubjektkontraste						
Quelle		df	F	Sig.	Partielles Eta-Quadrat	Beobachtete Aussagekraft (Observed Power)[a]
Selbsttrans-zendenz	Sphärizitätsannahme	1	1.694	.206	.069	.239
Selbsttrans-zendenz * Gruppe	Sphärizitätsannahme	1	6.217	.020	.213	.666
Fehler (Selbsttrans-zendenz)	Sphärizitätsannahme	23				

Deskriptive Statistik				
	Gruppe	Mittel	SD	N
Belohnungs-abhängigkeit T0	Intervention	49.17	10.116	12
	Kontrolle	48.00	15.050	13
	Gesamt	48.56	12.669	25
Belohnungs-abhängigkeit T_final	Intervention	50.00	12.606	12
	Kontrolle	46.46	14.892	13
	Gesamt	48.16	13.674	25

Test der Innersubjektkontraste						
Quelle		df	F	Sig.	Partielles Eta-Quadrat	Beobachtete Aussagekraft (Observed Power)[a]
Belohnungs-abhängigkeit	Sphärizitätsannahme	1	.018	.894	.001	.052
Belohnungs-abhängigkeit * Gruppe	Sphärizitätsannahme	1	.206	.654	.009	.072
Fehler (Belohnungs-abhängigkeit)	Sphärizitätsannahme	23				

FEEL-KJ (Fragebogen zur Emotionsregulation bei Kindern und Jugendlichen)

- Dateiname: Feel.xlsx
- Titel der Daten: Fragebogendaten
- Beschreibung der Daten: Die Daten sind in Karten für T-Scores und die erste Karte als aggregierter Score nach der Anzahl der Teilnehmer dargestellt.

Der FEEL-KJ wurde von den Jugendlichen vor Beginn der Studie, zwei Monate nach Studienbeginn sowie eine und 26 Wochen nach Abschluss des Interventionsprogramms beantwortet. Zu den vier Zeitpunkten gab es keine statistisch signifikanten Unterschiede zwischen der Kontroll- und Interventionsgruppe. Die ANOVA mit wiederholten Messungen ergab keine signifikanten zeitabhängigen Veränderungen bei den Emotionsregulationsstrategien der Interventions- oder Kontrollgruppe.

Deskriptive Statistik				
	Gruppe	Mittel	SD	N
Adaptive Strategien Gesamt T0	Intervention	41.83	10.573	12
	Kontrolle	41.77	13.516	13
	Gesamt	41.80	11.941	25
Adaptive Strategien Gesamt T3	Intervention	60.92	13.548	12
	Kontrolle	58.92	17.231	13
	Gesamt	59.88	15.284	25
Adaptive Strategien Gesamt T_final	Intervention	39.25	10.411	12
	Kontrolle	41.31	13.542	13
	Gesamt	40.32	11.936	25
Adaptive Strategien Gesamt T6M follow-up	Intervention	40.58	9.346	12
	Kontrolle	45.77	13.893	13
	Gesamt	43.28	11.981	25

Test der Innersubjektkontraste						
Quelle		df	F	Sig.	Partielles Eta-Quadrat	Beobachtete Aussagekraft (Observed Power)[a]
Adaptive Strategien Gesamt	Greenhouse-Geisser	1.268	13.463	.000	.369	.971
Adaptive Strategien Gesamt* Gruppe	Greenhouse-Geisser	1.268	.379	.592	.016	.096
Fehler (Adaptive Strategien Gesamt)	Greenhouse-Geisser	29.164				

Deskriptive Statistik				
	Gruppe	Mittel	SD	N
Maladaptive Strategien. Gesamt T0	Intervention	60.92	13.548	12
	Kontrolle	58.92	17.231	13
	Gesamt	59.88	15.284	25
Maladaptive Strategien. Gesamt T3	Intervention	59.00	13.045	12
	Kontrolle	59.15	17.497	13
	Gesamt	59.08	15.201	25
Maladaptive Strategien. Gesamt T_final	Intervention	62.75	10.964	12
	Kontrolle	60.77	14.208	13
	Gesamt	61.72	12.532	25
Maladaptive Strategien. Gesamt T6M follow-up	Intervention	59.25	15.028	12
	Kontrolle	55.08	12.977	13
	Gesamt	57.08	13.865	25

Test der Innersubjektkontraste						
Quelle		df	F	Sig.	Partielles Eta-Quadrat	Beobachtete Aussagekraft (Observed Power)[a]
Maladaptive Strategien Gesamt	Sphärizitätsannahme	3	2.070	.112	.083	.508
Maladaptive Strategien Gesamt * Gruppe	Sphärizitätsannahme	3	.444	.723	.019	.135
Fehler (Maladaptive Strategien Gesamt)	Sphärizitätsannahme	69				

Deskriptive Statistik				
	Gruppe	Mittel	SD	N
Maladaptive Strategien. Wut. T0	Intervention	59.50	13.879	12
	Kontrolle	60.31	16.276	13
	Gesamt	59.92	14.863	25
Maladaptive Strategien. Wut. T3	Intervention	59.33	13.839	12
	Kontrolle	59.85	14.194	13
	Gesamt	59.60	13.733	25
Maladaptive Strategien. Wut. T_final	Intervention	61.00	10.694	12
	Kontrolle	60.15	10.049	13
	Gesamt	60.56	10.153	25
Maladaptive Strategien. Wut. T6M follow-up	Intervention	57.33	16.053	12
	Kontrolle	53.15	14.053	13
	Gesamt	55.16	14.879	25

Test der Innersubjektkontraste						
Quelle		df	F	Sig.	Partielles Eta-Quadrat	Beobachtete Aussagekraft (Observed Power)[a]
Maladaptive Strategien. Wut	Sphärizitätsannahme	3	2.230	.092	.088	.542
Maladaptive Strategien Wut * Gruppe	Sphärizitätsannahme	3	.495	.687	.021	.146
Fehler (Maladaptive Strategien Wut)	Sphärizitätsannahme	69				

Deskriptive Statistik				
	Gruppe	Mittel	SD	N
Maladaptive Strategien. Angst T0	Intervention	60.50	12.703	12
	Kontrolle	56.31	18.553	13
	Gesamt	58.32	15.832	25
Maladaptive Strategien. Angst T3	Intervention	54.00	13.987	12
	Kontrolle	54.77	18.842	13
	Gesamt	54.40	16.350	25
Maladaptive Strategien. Angst T_final	Intervention	61.25	12.084	12
	Kontrolle	58.15	17.530	13
	Gesamt	59.64	14.936	25
Maladaptive Strategien. Angst T6M follow-up	Intervention	57.58	15.524	12
	Kontrolle	51.31	13.756	13
	Gesamt	54.32	14.673	25

Test der Innersubjektkontraste						
Quelle		df	F	Sig.	Partielles Eta-Quadrat	Beobachtete Aussagekraft (Observed Power)[a]
Adaptive Strategien. Angst	Sphärizitätsannahme	3	2.890	.042	.112	.666
Adaptive Strategien. Angst * Gruppe	Sphärizitätsannahme	3	.847	.473	.036	.225
Fehler (Adaptive Strategien. Angst)	Sphärizitätsannahme	69				

Deskriptive Statistik				
	Gruppe	Mittel	SD	N
Maladaptive Strategien. Traurigkeit T0	Intervention	60.00	10.514	12
	Kontrolle	57.46	15.634	13
	Gesamt	58.68	13.212	25
Maladaptive Strategien. Traurigkeit T3	Intervention	59.42	11.469	12
	Kontrolle	58.62	16.711	13
	Gesamt	59.00	14.145	25
Maladaptive Strategien. Traurigkeit T_final	Intervention	60.92	11.373	12
	Kontrolle	59.23	12.969	13
	Gesamt	60.04	12.005	25
Maladaptive Strategien. Traurigkeit T6M follow-up	Intervention	57.92	15.894	12
	Kontrolle	57.15	13.795	13
	Gesamt	57.52	14.529	25

Test der Innersubjektkontraste						
Quelle		df	F	Sig.	Partielles Eta-Quadrat	Beobachtete Aussagekraft (Observed Power)[a]
Maladaptive Strategien Traurigkeit	Sphärizitätsannahme	3	.424	.736	.018	.131
Maladaptive Strategien Traurigkeit * Gruppe	Sphärizitätsannahme	3	.069	.976	.003	.062
Fehler (Maladaptive Strategien Traurigkeit)	Sphärizitätsannahme	69				

Deskriptive Statistik				
	Gruppe	Mittel	SD	N
Adaptive Strategien Traurigkeit T0	Intervention	44.00	10.804	12
	Kontrolle	42.23	13.633	13
	Gesamt	43.08	12.134	25
Adaptive Strategien Traurigkeit T3	Intervention	38.75	10.746	12
	Kontrolle	41.69	13.022	13
	Gesamt	40.28	11.830	25
Adaptive Strategien Traurigkeit T_final	Intervention	41.08	10.031	12
	Kontrolle	44.62	12.784	13
	Gesamt	42.92	11.449	25
Adaptive Strategien Traurigkeit T6M follow-up	Intervention	44.25	9.206	12
	Kontrolle	42.85	13.403	13
	Gesamt	43.52	11.366	25

Test der Innersubjektkontraste						
Quelle		df	F	Sig.	Partielles Eta-Quadrat	Beobachtete Aussagekraft (Observed Power)[a]
Adaptive Strategien Traurigkeit	Greenhouse-Geisser	2.212	1.156	.327	.048	.254
Adaptive Strategien Traurigkeit * Gruppe	Greenhouse-Geisser	2.212	1.003	.381	.042	.225
Fehler (Adaptive Strategien Traurigkeit)	Greenhouse-Geisser	50.866				

Deskriptive Statistik				
	Gruppe	Mittel	SD	N
Adaptive Strategien Angst T0	Intervention	41.75	10.306	12
	Kontrolle	41.92	13.009	13
	Gesamt	41.84	11.546	25
Adaptive Strategien Angst T3	Intervention	39.50	9.959	12
	Kontrolle	43.38	13.345	13
	Gesamt	41.52	11.765	25
Adaptive Strategien Angst T_final	Intervention	42.92	10.335	12
	Kontrolle	48.38	13.920	13
	Gesamt	45.76	12.394	25
Adaptive Strategien Angst T6M follow-up	Intervention	46.92	8.867	12
	Kontrolle	45.00	11.825	13
	Gesamt	45.92	10.340	25

Test der Innersubjektkontraste						
Quelle		df	F	Sig.	Partielles Eta-Quadrat	Beobachtete Aussagekraft (Observed Power)[a]
Adaptive Strategien Angst	Sphärizitätsannahme	3	3.337	.024	.127	.735
Adaptive Strategien Angst * Gruppe	Sphärizitätsannahme	3	1.634	.189	.066	.411
Fehler (Adaptive Strategien Angst)	Sphärizitätsannahme	69				

SDQ (Fragebogen zu Stärken und Schwierigkeiten)

- Dateiname: SDQ.xlsx

- Titel der Daten: Fragebogendaten

- Beschreibung der Daten: Die Daten sind nach der Anzahl der Teilnehmer und der Gruppe, der sie angehören, sortiert und der Wert sowie der aggregierte Score sind in derselben Karte enthalten.

Der SDQ wurde von den Eltern der jüngeren Gruppe vor der Intervention, sechs Monate nach Beginn und zwei Wochen nach Abschluss der Studie ausgefüllt. Es gab keine Unterschiede zwischen der Interventions- und der Kontrollgruppe, und es war keine Veränderung im Zeitverlauf erkennbar. Die Werte eines Elternteils jeder Gruppe lagen über der Norm.

Deskriptive Statistik				
	Gruppe	Mittel	SD	N
SDQ_E_T0	Intervention	9.2	5.4	13
	Kontrolle	9.1	5.0	16
	Gesamt	9.16	5.1	29
SDQ_E_T6	Intervention	8.5	4.1	13
	Kontrolle	8.9	5.1	16
	Gesamt	8.8	4.6	29
SDQ_E_T_final	Intervention	6.8	4.1	13
	Kontrolle	7.8	5.4	16
	Gesamt	7.3	4.8	29

SDQ: Fragebogen zu Stärken und Schwierigkeiten; E: Eltern; t0: vor der Studie; t6: 6 Monate nach Beginn; t13: nach Beendigung der Studie

Test der Innersubjektkontraste						
Quelle		df	F	Sig.	Partielles Eta-Quadrat	Beobachtete Aussagekraft (Observed Power)[a]
SDQ	Greenhouse-Geisser	1.554	2.534	.103	.086	.423
SDQ * Gruppe	Greenhouse-Geisser	1.554	.157	.801	.006	.071
Fehler (SDQ)	Greenhouse-Geisser	41.955				

DIK (Depressionsinventar für Kinder und Jugendliche)

Der DIKJ wurde von der ersten Gruppe von Kindern vor, sechs Monate nach Beginn und eine Woche nach Abschluss der Studie ausgefüllt. Es gab keine Veränderungen im Zeitverlauf und keine Unterschiede in den Werten zwischen Interventions- und Kontrollgruppe. Zwei Kinder der Interventionsgruppe und eines der Kontrollgruppe wiesen Werte auf, die für eine Depression auffällig waren.

Deskriptive Statistik

	Gruppe	Mittel	SD	N
DIKJ_T0	Intervention	8.0833	5.68024	12
	Kontrolle	7.0000	7.36659	16
	Gesamt	7.4643	6.60237	28
DIKJ_T6	Intervention	5.7500	5.47930	12
	Kontrolle	5.1250	3.61248	16
	Gesamt	5.3929	4.42501	28
DIKJ_T_final	Intervention	7.5833	5.77547	12
	Kontrolle	5.4375	4.32001	16
	Gesamt	6.3571	5.01268	28

DIKJ: Depressionsinventar; tO: vor der Studie; t6: 6 Monate nach Beginn; t13: nach Beendigung der Studie

Test der Innersubjektkontraste						
Quelle		df	F	Sig.	Partielles Eta-Quadrat	Beobachtete Aussagekraft (Observed Power)[a]
DIKJ	Sphärizitätsannahme	2	1.854	.167	.067	.369
DIKJ * Gruppe	Sphärizitätsannahme	2	.255	.776	.010	.088
Fehler (DIKJ)	Sphärizitätsannahme	52				

CHIP-D (Coping Health Inventory für Eltern)

- Dateiname: Chip-D.xIsx

- Titel der Daten: Fragebogendaten

- Beschreibung der Daten: Die Daten sind nach der Anzahl der Teilnehmer auf zwei Karten sortiert. Die zweite Karte (Messwdh) zeigt die Daten der anderen Messzeitpunkte.

Der CHIP wurde von den Eltern der jugendlichen Gruppe vor und am Ende des Interventionsprogramms beantwortet.

Vor Beginn der Studie zeigten die Eltern der Interventionsgruppe niedrigere Scores für CHIP-FAM (p=0.016) und CHIP-ALL (p=0.022) als die Eltern der Kontrollgruppe.

Eltern von Kindern aus dem Interventionsprogramm hielten soziale Unterstützung am Ende der Studie für wichtiger als vor der Befragung (p=0.046). Die Scores (Perzentile) für CHIP-FAM (p=0.036), CHIP-MED (p=0.058) und CHIP-ALL (p=0.066) der Eltern aus der Kontrollgruppe waren am Ende der Studie niedriger als zu Beginn der Befragung. Am Ende der Studie wiesen die Eltern beider Gruppen ähnliche Scores auf.

Die meisten Eltern der Interventionsgruppe bewerteten die soziale Unterstützung zu beiden untersuchten Zeitpunkten im Vergleich zur Normgruppe als durchschnittlich oder überdurchschnittlich. Familiäre und medizinische Unterstützung wurden als weniger nützlich angesehen. Die Eltern der Kontrollgruppe hielten die soziale Unterstützung und im Gegensatz zu den Eltern der Interventionsgruppe wurde die familiäre Unterstützung vor der Studie als durchschnittlich und überdurchschnittlich bewertet. Am Ende der Studie wurden die familiäre und medizinische Unterstützung als unterdurchschnittlich eingestuft, die Nützlichkeit der sozialen Unterstützung blieb überdurchschnittlich.

Deskriptive Statistik

	Gruppe	Mittel	SD	N
CHIPFAM T0	Intervention	27.950	19.6650	12
	Kontrolle	57.731	29.9980	13
	Intervention	43.436	29.2878	25
CHIPFAM T_final	Intervention	34.208	32.0754	12
	Kontrolle	39.715	32.9682	13
	Gesamt	37.072	31.9825	25

Test der Innersubjektkontraste						
Quelle		df	F	Sig.	Partielles Eta-Quadrat	Beobachtete Aussagekraft (Observed Power)[a]
CHIPFAM	Sphärizitätsannahme	1	1.527	.229	.062	.220
CHIPFAM * Gruppe	Sphärizitätsannahme	1	6.509	.018	.221	.686
Fehler (CHIPFAM)	Sphärizitätsannahme	23				

Deskriptive Statistik				
	Gruppe	Mittel	SD	N
CHIPSUP T0	Intervention	60.250	24.6254	12
	Kontrolle	71.369	18.9163	13
	Gesamt	66.032	22.1133	25
CHIPSUP T_final	Intervention	68.525	19.8606	12
	Kontrolle	66.908	25.7882	13
	Gesamt	67.684	22.6712	25

Test der Innersubjektkontraste						
Quelle		df	F	Sig.	Partielles Eta-Quadrat	Beobachtete Aussagekraft (Observed Power)[a]
CHIPSUP	Sphärizitätsannahme	1	.238	.630	.010	.075
CHIPSUP * Gruppe	Sphärizitätsannahme	1	2.653	.117	.103	.345
Fehler (CHIPSUP)	Sphärizitätsannahme	23				

Deskriptive Statistik				
	Gruppe	Mittel	SD	N
CHIPMED T0	Intervention	28.642	28.4827	12
	Kontrolle	45.338	29.8804	13
	Gesamt	37.324	29.8451	25
CHIPMED T_final	Intervention	28.617	23.2432	12
	Kontrolle	29.885	30.4210	13
	Gesamt	29.276	26.6599	25

Test der Innersubjektkontraste						
Quelle		df	F	Sig.	Partielles Eta-Quadrat	Beobachtete Aussagekraft (Observed Power)[a]
CHIPMED	Sphärizitätsannahme	1	1.651	.212	.067	.234
CHIPMED * Gruppe	Sphärizitätsannahme	1	1.640	.213	.067	.233
Fehler (CHIPMED)	Sphärizitätsannahme	23				

Deskriptive Statistik				
	Gruppe	Mittel	SD	N
CHIP T0	Intervention	35.067	26.5818	12
	Kontrolle	60.038	24.7113	13
	Gesamt	48.052	28.1303	25
CHIP Gesamt T_final	Intervention	42.225	32.5943	12
	Kontrolle	46.100	31.1859	13
	Gesamt	44.240	31.2588	25

Test der Innersubjektkontraste						
Quelle		df	F	Sig.	Partielles Eta-Quadrat	Beobachtete Aussagekraft (Observed Power)[a]
CHIP Gesamt	Sphärizitätsannahme	1	.501	.486	.021	.104
CHIP Gesamt * Gruppe	Sphärizitätsannahme	1	4.850	.038	.174	.560
Fehler (Gesamt)	Sphärizitätsannahme	23				

Bewertung des Interventionsprogramms (eo ipso-Fragebogen), Jugendliche

Im eo ipso Fragebogen bewerteten alle Jugendlichen (n=12) das Interventionsprogramm als positiv und berichten einen erheblichen positiven Einfluss auf die Psyche. Die Interaktion mit den Hunden, Gleichaltrigen, mit den Hundetrainern und anderen Betreuern wurde von den meisten Jugendlichen als Die angenehm empfunden (Kontakt zu Hunden, Gleichaltrigen, Personal: n=6; nur Hunde: n=2; nur Gleichaltrige n=1). Die meisten Jugendlichen stellen positive Emotionen wie Freude, Aufregung, Vergnügen, Selbstwertgefühl, Verantwortungsbewusstsein und Gemeinschaftsgefühl fest (n=12). Eine Teilnehmerin verlor vorübergehend ihre Hundephobie. Veränderungen des Schmerzempfinden und positive Auswirkungen auf die körperliche Leistungsfähigkeit wurden nur von einer Teilnehmerin erwähnt, aber die meisten Teilnehmer beantworteten diesen Abschnitt des Fragebogens nicht.

Fünf Mädchen erwähnten, dass die Treffen zu selten stattfanden, dass sie zu wenig Möglichkeiten hatten, mit den Hunden in Kontakt zu kommen und dass es draußen manchmal sehr kalt war.

Vier Eltern bemerkten Veränderungen in der Schmerzwahrnehmung und im Umgang mit Schmerzen ihrer Jugendlichen, sieben erwähnten positive Auswirkungen der Intervention, z. B. auf das Selbstwertgefühl, die Offenheit, die Freude, und dass der Kontakt mit Kindern, die ebenfalls unter chronischen Schmerzen litten, einen positiven Einfluss auf ihre Kinder hatte.

Weiterführende Diskussion der zusätzlichen Ergebnisse

Emotionsregulation, Stimmung und Verhalten

Der FEEL-Fragebogen wurde eingesetzt, um Ressourcenprofile, die Entwicklung von Emotionsregulationsstrategien, psychosoziale Kompetenzen, Stresswahrnehmung und -bewältigung zu ermitteln und den Fortschritt während des Interventionsprogramms zu messen.

Wir konnten keinen Einfluss der Intervention auf die Emotionsregulationsstrategien feststellen. Dies lässt sich dadurch erklären, dass chronische Schmerzen zu einer Veränderung der Schmerzempfindlichkeit, der Schmerzwahrnehmung und einer veränderten Verarbeitung von Belohnungsreizen führen. Diese Veränderungen erfolgen durch operantes Lernen, was die Behandlung chronischer Schmerzen erschwert, da ein erneuter Lernprozess stattfinden muss, der jedoch durch einen permanent erregten Zustand gehemmt wird [21]. Im Gegensatz dazu wurden bei Kindern mit Autismus-Spektrum-Störung die soziale Annäherung und die pro-sozialen Fähigkeiten verbessert und der Rückzug verringert, wenn Meerschweinchen im Klassenzimmer gehalten wurden und die Kinder die Möglichkeit hatten, 20 Minuten lang mit den Tieren zu interagieren [7].

Wie die Ergebnisse des FEEL-Fragebogens zeigen, wiesen bis zu 50 % der Jugendlichen aus der Interventions- und aus der Kontrollgruppe eine unterdurchschnittliche adaptive Emotionsregulation (z. B. Problemlösung. Akzeptanz, Humorverstärkung) und häufiger maladaptive Emotionsregulationsstrategien (z. B. sozialer Rückzug, Selbstabwertung, Grübeln) auf. Diese Ergebnisse stehen im Einklang mit der Hypothese, dass die Entwicklung chronischer Schmerzen auf assoziativem Lernen beruht und von maladaptiven aversiven Gedächtnis- und Extinktionsprozessen abhängt, die zu Angst vor Schmerzen und Vermeidungsstrategien, einschließlich Inaktivität und sozialem Rückzug, führen [21].

Der JTI wurde verwendet, um automatische emotionale Reaktionen zu analysieren, die verschiedene Persönlichkeitsstile charakterisieren (Temperamentsskalen), und um die persönliche Reife zu beurteilen, die durch zentrale Selbstkonzepte gekennzeichnet ist (Charakterskalen). Die Intervention hatte keinen Einfluss auf die Ergebnisse der Temperament- und Charakterskalen im Vergleich zu den Kontrollen. Nach Cloninger ist das Temperament angeboren, kann aber durch den Charakter moduliert werden. Der Charakter entwickelt sich durch soziokulturelle Lernprozesse und wird durch das Temperament beeinflusst [34].

Etwa 40 % der Teilnehmer an dieser Studie wiesen eine verminderte persönliche Reife und eine geringere Selbststeuerung auf, die für einen „unsicher-ineffektiven“ Charakterstil typisch sind (z. B. hilflos, unzufrieden und ziellos). Fast die Hälfte der Jugendlichen aus der Interventions- und Kontrollgruppe wies einen „stoisch-mitleidigen" und/oder „vorsichtig-ängstlichen" Persönlichkeitsstil auf (z. B. uninteressiert, unbeholfen, pessimistisch, zweifelnd, unsicher, schwach, schüchtern). Die Kombination aus geringer Neuheitssuche und hoher Schadensvermeidung charakterisiert den Temperamentstyp „starr-introvertiert“, der bei einem Viertel der Teilnehmer zu beobachten war (passiv, introvertierte Wut, Suche nach Sicherheit und Ruhe). Eine kürzlich durchgeführte Untersuchung bei Kindern mit Migräne ergab ebenfalls eine hohe Prävalenz von

Schadensvermeidung und Hartnäckigkeit sowie geringere Werte bei der Neuheitssuche. Auch die Prävalenz der Selbststeuerung war bei Kindern mit Migräne geringer als bei unseren Daten [35]. Auch Patienten mit chronischen Schmerzen, die nicht mit Migräne in Verbindung gebracht werden (z. B. periphere Neuropathie, Radikulopathien, Osteoarthritis), wiesen hohe Werte für Schadensvermeidung und niedrige Werte für Neuheitssuche, Selbststeuerung und Kooperationsbereitschaft auf [36]. Die Schadensvermeidung ist eine vererbte Prädisposition für frühe Angst und führt zu einer automatischen Hemmung, z. B. kann die Angst vor Schmerzen automatisch zu Vermeidungsverhalten führen [37]. Ein höherer Wert für Schadensvermeidung könnte daher auf chronische Schmerzen zurückzuführen sein, könnte aber auch die Entwicklung chronischer Schmerzen begünstigen [37]. Es wird angenommen, dass Vermeidungsverhalten ein zentraler Aspekt von chronischen Schmerzen ist. Schmerz, Angst, Furcht, chronische Schmerzen und Vermeidungsverhalten bedingen sich gegenseitig. Die Inkongruenz von emotionalem und sensorischem Schmerz, bei der der schmerzhafte Reiz nachlässt, Angst und Vermeidung aber bestehen bleiben, führte zur Entwicklung des Angst-Vermeidungs-Modells der übersteigerten Schmerzwahrnehmung. Das Modell der Bewegungsangst basiert auf der Erwartung, dass wiederkehrende Schmerzen aufgrund von Bewegung zu einem erhöhten Leidensdruck und zu Behinderungen führen [38, 39]. Angst und Vermeidung tragen zum Fortbestehen des Schmerzverhaltens und der Behinderung bei. Es entwickelt sich ein Teufelskreis aus katastrophisierendem Schmerz, Angst und Sicherheitsverhalten [37], und schließlich können Angst, Erwartung und Überschätzung des Schmerzes zu einer stärkeren Behinderung führen als der Schmerz selbst [38].

Mit der CBCL werden verhaltensbezogene, emotionale und somatische Abnormitäten ermittelt. Die Intervention änderte weder die Kompetenz- noch die Syndromskalen-Scores, wie sie von der CBCL erfasst werden, aber die Werte für Externalisierung, Internalisierung und die Gesamt-Syndromskalen-Scores waren nach 26 Wochen niedriger als unmittelbar nach der Intervention.

Abgesehen von somatischen Beschwerden waren die Internalisierungs- und Rückzugswerte in beiden Gruppen von Jugendlichen häufig klinisch. Die Gesamt-Scores und die ängstlichen/depressiven Scores waren weniger häufig klinisch. Rückzug wird als dysfunktionales, maladaptives Vermeidungsverhalten interpretiert, das zu Angst, Behinderung und Schmerzpersistenz führt.

Interessanterweise zeigte sich bei der elterlichen Einschätzung ein geringerer Prozentsatz an ängstlich-depressivem Verhalten (16 %) und weniger sozialer Rückzug (23 %) als bei der Selbsteinschätzung im JTCI. Fast die Hälfte der Jugendlichen zeigte erhöhte JTCI-Werte in Bezug auf den unsicher-ineffektiven und den vorsichtigen ängstlichen Persönlichkeitsstil. Diese Abweichung könnte mit Schmerzkatastrophisierung, erhöhter Angst, Schmerzsensibilisierung oder verringerten Schmerzschwellen zusammenhängen, die bei Jugendlichen mit chronischen Schmerzen auftreten [39]. Darüber hinaus kommt es aufgrund aversiver Stimuli zu einer Verzerrung in Richtung negativer Affekte und Motivation [21].

Externe Einflüsse

Ein Fragebogen wurde entworfen, um mögliche externe Faktoren zu bewerten, die das Studienergebnis beeinflusst haben könnten. Die Fragen wurden entweder mit „ja“ oder „nein“ beantwortet und enthielten vertiefende Freitextfelder. Die Fragen lauteten:

1. Änderungen der Medikation, die zu einer Besserung der Erkrankung führten

2. Neu angebotene Unterstützung

3. Eltern erhielten neue Unterstützung

4. Bedeutsame negative Zufälle

5. Bedeutsame positive Einflüsse

Der Fragebogen wurde von den Jugendlichen der Interventions- und Kontrollgruppe eine und 26 Wochen nach Abschluss der Studie ausgefüllt.

Verweise auf das ergänzende Material

Achenbach TM. Manual for the child behavior check-list/4-18 and 1991 profile. Burlington, VT: University of Vermont Department of Psychiatry; 1991.

Goodman R. The Strengths and Difficulties Questionnaire: a research note. J Child Psychol Psychiatry. 1997;38(5):581-586. DOI: 10.1111/j.1469-7610.1997.tb01545.x

Goodman R, Ford T, Corbin T, Meltzer H. Using the Strengths and Difficulties Questionnaire (SDQ) multi-informant algorithm to screen looked-after children for psychiatric disorders. Eur Child Adolesc Psychiatry. 2004;13 Suppl 2:II25-II31. DOI: 10.1007/s00787-004-2005-3

Goth K, Schmeck K. Das Junior Temperament und Charakter Inventar. Göttingen: Hogrefe; 2009.

Grob A, Smolemski C. Fragebogen zur Erhebung der emotionsregulation bei Kindern und Jugendlichen (FEEL-KJ). Bern: Hans Huber Verlag; 2009.

Naab S, Hauer M, Vorderholzer U, Hautzinger M. [Depressive disorders in juveniles: diagnosis and therapy]. Fortschr Neurol Psychiatr. 2015;83(1):49-62. DOI: 10.1055/s-0034-1385776

Woerner W, Becker A, Friedrich C, Klasen H, Goodman R, Rothenberger A. [Normal values and evaluation of the German parents' version of Strengths and DIfficulties Questionnaire (SDQ): Results of a representative field study]. Z Kinder Jugendpsychiatr Psychother. 2002;30(2):105-112. DOI: 10.1024//1422-4917.30.2.105
